# Dye label interference with RNA modification reveals 5-fluorouridine as non-covalent inhibitor

**DOI:** 10.1093/nar/gku908

**Published:** 2014-10-09

**Authors:** Felix Spenkuch, Gerald Hinze, Stefanie Kellner, Christoph Kreutz, Ronald Micura, Thomas Basché, Mark Helm

**Affiliations:** 1 Institute of Pharmacy and Biochemistry, University of Mainz, Staudingerweg 5, D-55128 Mainz, Germany; 2 Institute of Physical Chemistry, University of Mainz, Duesbergweg 10–14, D-55128 Mainz, Germany; 3 Institute of Organic Chemistry, Center for Molecular Biosciences (CMBI), University of Innsbruck, Innrain 52A, A-60230 Innsbruck, Austria; 4 Institute of Organic Chemistry, Center for Chemistry and Biomedicine - CCB, University of Innsbruck, Innrain 80/82, A-60230 Innsbruck, Austria

## Abstract

The interest in RNA modification enzymes surges due to their involvement in epigenetic phenomena. Here we present a particularly informative approach to investigate the interaction of dye-labeled RNA with modification enzymes. We investigated pseudouridine (Ψ) synthase TruB interacting with an alleged suicide substrate RNA containing 5-fluorouridine (5FU). A longstanding dogma, stipulating formation of a stable covalent complex was challenged by discrepancies between the time scale of complex formation and enzymatic turnover. Instead of classic mutagenesis, we used differentially positioned fluorescent labels to modulate substrate properties in a range of enzymatic conversion between 6% and 99%. Despite this variegation, formation of SDS-stable complexes occurred instantaneously for all 5FU-substrates. Protein binding was investigated by advanced fluorescence spectroscopy allowing unprecedented simultaneous detection of change in fluorescence lifetime, anisotropy decay, as well as emission and excitation maxima. Determination of *K*_d_ values showed that introduction of 5FU into the RNA substrate increased protein affinity by 14× at most. Finally, competition experiments demonstrated reversibility of complex formation for 5FU-RNA. Our results lead us to conclude that the hitherto postulated long-term covalent interaction of TruB with 5FU tRNA is based on the interpretation of artifacts. This is likely true for the entire class of pseudouridine synthases.

## INTRODUCTION

The canon of known nucleoside building blocks that occur naturally in nucleic acids has rapidly expanded in the last two decades (1,2,3). Nucleoside modifications owe their recent surge in popularity to their involvement in epigenetic phenomena, both DNA and RNA related, which have been uncovered by newly emerging technologies, in particular by deep sequencing approaches (4). Consequently, there is a renewed interest in modification enzymes and their interaction with nucleic acids.

This applies particularly to the interaction of RNA and pseudouridine synthases (Pus) (5,6), which have been investigated for decades, although key features, such as, e.g. catalytic mechanism (7,8), remain elusive. The catalytic product of Pus is pseudouridine (Ψ), a C-glycosidic isomer of uridine, which stands out in the crowd of >150 RNA modifications. Most striking among the many functions and locations (9–13) of this most abundant RNA modification (14) is the recent observation of its capability to alter message decoding (15,16) and the recent discovery of its wide-spread distribution in eukaryotic mRNAs (17,^18^), putting it square in the bulls eye of epigenetic research along with, e.g. m^6^A (19,20).

In this backdrop, studies on the interaction of Pus enzymes with target RNA carrying 5-fluorouridine (5FU) as a substrate analog suffer from contradictory reports in the literature. As in the interaction of 5FU with thymidylate synthase (21), 5FU was promoted to undergo a covalent suicide interaction with Pus enzymes when placed *en lieu* of the target uridine (22). This paradigm is especially prevalent for the TruB (Pus4) class of Pus enzymes, where the suicide adduct was reported to be detectable by Sodium dodecyl sulfate-polyacrylamide gel electrophoresis (SDS-PAGE) (23). This complex was investigated using a 5FU containing T stem-loop (TSL) minimal substrate (24), which was also used for crystallography (23,25–26). Isomerization to a supposed intermediate was evident from cocrystals of protein and RNA (23,25,27). Similar isomerization reactions could be proven biochemically for several Pus (8,28–31). However, kinetic analysis failed to detect any inhibitory effects of 5FU on TruB and consequently put in doubt the central role of 5FU in the field (32). Importantly, a thorough inspection of the literature reveals that all crystallographic data could potentially be reconciled by alternative mechanisms, but that the above SDS-stable complex is the only tangible piece of experimental evidence for a true, namely, stable, suicide interaction.

Research in the Pus field is hampered by shortcomings in the traditionally powerful mutagenesis approaches. Besides the catalytic aspartate, only an arginine residue could be identified as important for catalysis (33). Of note, this is in stark contrast to other enzyme classes, where suicide mechanisms of 5-fluoropyridines (21,34–40) have been proven by a variety of approaches, including trapping and investigation of the covalent intermediate (39,41–46). Mutagenesis of the RNA substrate has revealed recognition of nucleotides adjacent to the target uridine for Pus7 (47), but for TruB the essential recognition appears to be the 3D structure of the TSL, rather than specific nucleotides (24,25,27). We therefore decided to develop an alternative approach to generate tRNAs, that are weak substrates for Ψ synthase TruB from *Thermotoga maritima* (*tm*TruB), namely, by attaching fluorescent labels at positions known or anticipated to interfere with binding.

We observed formation of SDS-resistant complexes whose time scale and yields were incompatible with enzymatic turnover data. In-depth characterization of binding affinities and chase experiments by fluorescence-based techniques ruled out a covalent nature of the TruB-5FU complex and question the role of 5FU as inhibitor.

## MATERIALS AND METHODS

All chemicals were obtained from Carl Roth, Karlsruhe, Germany, if not mentioned otherwise.

### Oligonucleotide synthesis

The oligonucleotide carrying the 5-fluorouridine residue was synthesized in the group of Ronald Micura in a similar way as described previously (48), see Supplementary Figure S7 for MS (mass spectrometry) analysis. All other oligonucleotides, including the ones labeled with Cy5-NHS-Esters at 5-Aminohexyl-3-acrylimido-deoxycytidine (C49) or 5-Aminohexyl-3-acrylimido-uridine (U33) were purchased from IBA Göttingen, Germany (see Supplementary Table S2 for sequences).

### RNA synthesis

Transcription was carried out as described previously (49). For ligation a previously published protocol (50) was modified: Oligo nucleotides were 8 μM and the reaction was carried out with both T4 DNA ligase (Fermentas, St. Leon Roth, Germany) and in-house prepared T4 RNA ligase 2. To avoid dye bleaching ligation product bands were excised from preparative gels according to visual inspection and rechecked on analytical gels scanned on a GE Healthcare Typhoon 9400 for Cy5 (excitation 633 nm, emission 670BP30).

### Protein synthesis

An *Escherichia coli* Rosetta (DE3) pLysS strain (Merck Novagen, Darmstadt, Germany), containing a lac-operon controlled pET28 vector encoding C-terminal His_6_-tagged *Thermotoga maritima* *(T. maritima)* TruB, was used for protein expression. Cultures were grown in LB-Medium (Lennox) containing 50 μg/μl Kanamycin and 34 μg/μl Chloramphenicol and induced at OD_600_ = 0.6 with 0.5 mM IPTG (Isopropyl β-D-1-thiogalactopyranoside). Harvested cell pellets were resuspended in 100 mM Tris (tris(hydroxymethyl)aminomethane) pH 8.0, lysed with lysozyme and centrifuged (30 min 20 000 *g*, 4°C). The supernatant was boiled at 70°C for 20 min, centrifuged again. From the supernatant the protein was captured on a HisTrap HP Ni^2+^-NTA column (GE Healthcare, Munich, Germany) using a linear gradient up to 500 mM imidazole. Reconcentrated protein was changed into 500 mM KCl, 50 mM Tris-HCl pH 7.5 buffer (both by using Sartorius Vivaspin filters with 10 kDa cutoff (VWR, Darmstadt, Germany) and passed through a Superdex 200 10/300 GL column (GE Healthcare, Munich, Germany) using the same buffer. Final buffer after reconcentration was 1× microscale thermophoresis (MST) buffer (20 mM Tris-HCl, pH 7.5, 60 mM KCl, 0.02% Tween-20 (Sigma Aldrich, Taufkirchen, Germany)). Concentration was determined by Nanodrop (Thermo Fisher Scientific, Dreieich, Germany) and aliquots were flash frozen in liquid nitrogen and stored at –20°C.

### Turnover experiments and LC-MS/MS

Methods are detailed in the Supporting Information and experimental data is given in Supplementary Figure S8 and Table S3. Briefly, 1 μM RNA was folded by heating for 4 min at 75°C in water and cooling to room temperature in 15 min after addition of 5x MST buffer to 1x MST buffer final. The enzymatic reaction was carried out by incubation with 2 μM TruB for 70 min at 80°C. Enzyme was denatured by 10 min boiling at 95°C, RNA was digested, dephosphorylated and quantified via LC-MS/MS (liquid chromatography coupled to tandem mass spectrometry) using an internal standard (51).

### Gel shift experiments

Prior to enzyme addition tRNA was folded by heating for 4 min at 75°C in water and cooling to room temperature in 15 min after addition of MST buffer to 1x MST buffer final. All reactions were incubated for the times indicated, following 5 min incubation in 0.5× the respective loading dye at either 25°C or 95°C. All gels, either 20 × 30 cm 10% 8 M urea PAGE or 20 × 10 cm 10% SDS-PAGE with 6% stacking gel, were run at 100 V and room temperature. Gels were first scanned with a GE Healthcare Typhoon 9400 for Cy5 (excitation 633 nm, emission 670BP30) and then stained with SYBR Gold (Invitrogen), scanned for SYBR Gold (excitation 488 nm, emission 520BP40) and, if indicated, FRET (Förster resonance energy transfer) from SYBR Gold to Cy5 (excitation 488 nm, emission 670BP30), followed by Coomassie G-250 staining.

### MST experiments

Prior to enzyme addition tRNA was folded by heating for 4 min at 75°C in water and cooling to room temperature in 15 min after addition of MST buffer. Aliquots of RNA were titrated with an equal volume *tm*TruB from 1:1 dilutions in MST 1× buffer to a final concentration of 50 nM tRNA. Measurements were performed with a Nanotemper Monolith NT.115 (Nanotemper, Munich, Germany) after 30 min incubation time using standard treated capillaries, where every capillary represented a specific titration point. Settings were: Red excitation and detection, 70% LED, 40% MST power and 25°C temperature control setting. One experimental set consisted of titration of all four constructs followed by chase experiments on the same samples with unlabeled tRNA^Phe^ transcript. For chase experiments an aliquot of the titration series was supplemented with a final concentration of 3.3 μM unlabeled transcript (U55-tRNA, resulting in 35 nM, instead of 50 nM labeled tRNA concentration), incubated for 20 min and measured. The resulting data was analyzed using ‘NT analysis 1.4.27’. Further analysis and fitting to a 1:1one-to-one binding model (52) was carried out in Origin (OriginLab) by fitting all three data sets of a given construct at once to obtain the *K*_d_ value with the standard error of the fit.

### Time-resolved fluorescence

Folding of tRNA was achieved by heating for 4 min at 75°C in water and cooling to room temperature in 15 min after addition of MST buffer. Experiments were performed using 50 μl 200 nM tRNA solutions in 50 μl Quartz cuvette (Hellma analytics, Müllheim, Germany) employing a Fluorolog-3 spectrofluorometer (HORIBA Jobin-Yvon, Bensheim, Germany). Emission spectra were recorded upon excitation at 647 nm from 651 to 690 nm (increment 1 nm, slits at excitation 3 nm and at emission 1 nm). Excitation spectra were recorded at 668 nm while exciting from 630 to 663 nm (increment 1 nm, slits at excitation 1 nm and at emission 3 nm). Fluorescence lifetime measurements have been performed with the same spectrometer using a pulsed Fianium laser (Fianium, Southampton, United Kingdom, Sc400_2PP, 20 MHz) for excitation at 647 nm. A single photon detector (PMA Hybrid 50, PicoQuant, Berlin, Germany) in conjunction with a PicoHarp 300 module (PicoQuant, Berlin, Germany) allowed for time correlated single photon counting. For every titration point photons were collected in histogram mode up to a peak maximum of 65 000 counts. Two different polarization geometries VV and VH have been used to separate fluorescence lifetime from molecular reorientation effects. In chase experiment 45 μl free tRNA was measured, the sample was supplemented with 10 μl TruB (5.23 μM final concentration, 160 nM final concentration labeled tRNA), resulting in >90% binding, measured again and supplemented with 2 μl transcript solution (5.75 μM final concentration, 157.8 nM final concentration labeled tRNA) that was equally measured.

The resulting data were analyzed using self-written scripts in Matlab (MathWorks) and Origin (OriginLab) and fitted in Origin using a self-normalizing one-to-one binding-model (52), error bars represent the standard error of the fit as reported by Origin. Intensity decay data was fitted globally to extract lifetime and anisotropy data for every titration point, thereby generating a titration curve. For spectra titration curves were generated by fitting the spectral maxima to a single Gaussian.

## RESULTS

### LC-MS/MS and the gel shift band analysis

**Figure 1.**
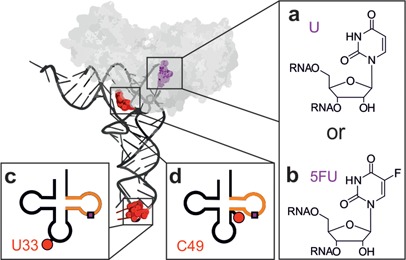
Tertiary (above) and secondary (below) structure representations of the yeast tRNA^Phe^ constructs. The substrate position 55 (purple) was either a U55 (**a**) or a 5FU55 (**b**). The Cy5-label (red) was either attached at position U33 outside the minimal substrate (orange) (**c**) or at position C49 inside the minimal substrate (**d**). The enzyme TruB bound to the tRNA as predicted by a docking model (27) is shown in gray.

**Figure 2.**
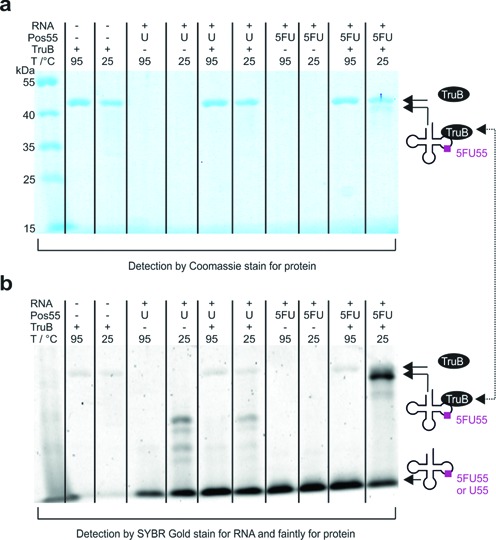
Analysis of SDS-stable complex formation of TruB with 5FU55- or U55-tRNA, respectively. Following 1 h incubation at 1 μM concentration and 70°C the complex was incubated with 0.5 vol SDS buffer for 5 min either at 25°C or at 95°C. **(a)** Coomassie stain for protein and **(b)** SYBR Gold stain for RNA. Note that SYBR Gold stains TruB faintly.

We chose yeast (*Saccharomyces cerevisiae*) tRNA^Phe^, a gold standard in the field, as substrate, whose structure and function have been characterized in abundance (53,54). In addition to the unmodified *in vitro* transcript (U55-tRNA), a tRNA carrying 5FU at substrate position 55 (5FU55-tRNA) was synthesized by molecular surgery techniques combining synthetic oligomers and ligation techniques (55,56) (Figure 1). Complexation experiments of *T. maritima* TruB with these constructs were performed at 70°C, near the enzyme's temperature optimum (54,57). Analysis by SDS-PAGE showed an SDS-stable complex in ∼40% yield, which was specific for 5FU55 (Figure 2) and could be disrupted by heating to 95°C in SDS loading dye.

This complex essentially reproduces a previous report using a 5FU-containing TSL minimal substrate (shown in orange in Figure 1), then interpreted as a covalent complex resulting from suicide action of 5FU (23). Surprisingly, attempts to characterize the kinetics of complex formation turned out ∼30% yield of complex already after 1 min of incubation in reaction buffer (*vide infra*). As Pus proteins, such as yeast Pus1p (58) or *E. coli* TruA, TruB and RIuA (6), are known to be slow enzymes, this fast complex formation is clearly at odds with the interpretation of the SDS-stable complex band as a covalently trapped catalytic intermediate. Since substrate turnover should correlate with formation of the allegedly trapped catalytic intermediate, we set out to generate substrates with variegated turnover yields.

Relevant recognition elements of TruB, as determined by traditional mutagenesis, were reported to be restricted to the TSL (orange in Figure 1) and form the key signature of T-loop structure (24). To keep the local TruB recognition structure near the turnover site intact, we made use of the finding that TSL and full tRNA are equally good substrates (24). A docking model of the x-ray structure of TruB to tRNA (27) (Figure 1) suggests binding exclusively to the TSL part of the tRNA L-shape. To generate substrates of variegated efficiency, we placed the cyanine dye Cy5 at two presumed strategic positions, namely, at U33, distant from the modification site in the anticodon loop, or near the modification site at the TSL-edge C49 (red dots/spheres in Figure 1), respectively. Correspondingly labeled tRNA molecules were synthesized in U55 and 5FU55 variants by splinted ligation, expecting moderate interference from the C49 label and no interference from the U33 label.

**Figure 3.**
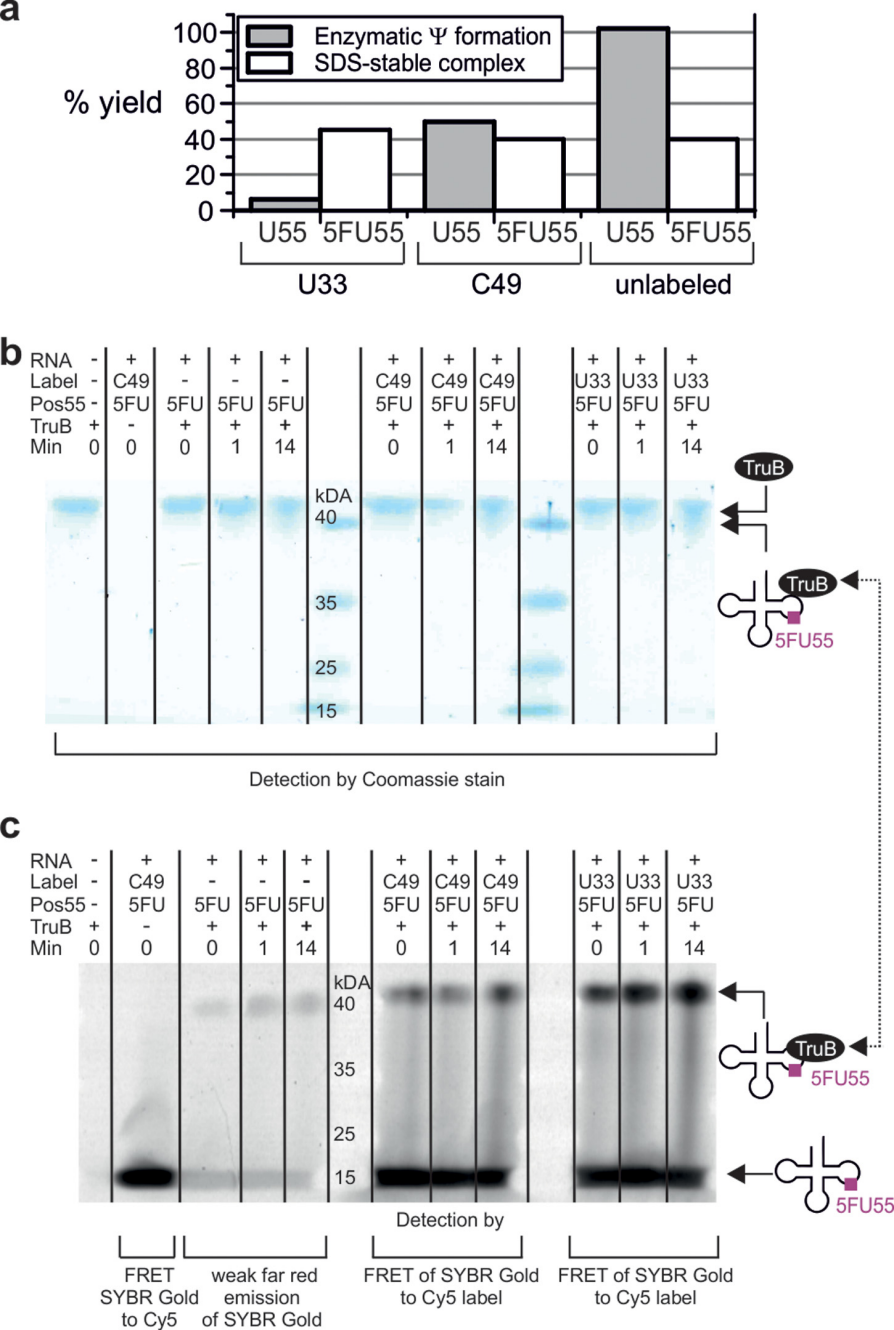
**(a)** Yield of Ψ generation in U55-tRNAs (assessed by LC-MS/MS in duplicate) compared to yield of SDS-stable complex by 5FU55-tRNAs (data quantified from b). **(b** and **c)** Kinetics of TruB binding to labeled and unlabeled 5FU55-tRNA analyzed by SDS-PAGE. Samples were incubated at 25°C for the time indicated and 5 min in 0.5 vol. SDS buffer prior to gel loading. Coomassie stain for protein (b), SYBR Gold stain for RNA (c) (excitation 488 nm, emission 670BP30). FRET from SYBR Gold to Cy5 generates the stronger signal of labeled tRNAs.

Substrate properties were analyzed in duplicate by LC-MS-based detection of pseudouridine after incubation with enzyme. For the C49-label, the expected inhibitory effect was confirmed: while the unlabeled transcript was turned over to >99%, C49-U55 was turned over to only ∼50% (Figure 3a). Surprisingly, the remote U33 label caused modification to only ∼6%, presumably as a consequence of long distance intramolecular rearrangement within the tRNA (59,60). While the low yield was unexpected, this weak substrate yield allowed to draw strong conclusions as to the nature of the SDS-stable complex, because it represented an inactive RNA containing all recognition elements of the fully active substrate. Logically, one would expect a trapped catalytic intermediate of the respective 5FU55-tRNA to form at reduced yield for the C49-tRNA, and the presence of the U33-label should essentially ablate formation of the covalent complex. However, as shown in Figure 3, all three 5FU55-tRNAs form substantial amounts of SDS-stable complex within seconds of mixing at 25°C.

In the particular case of the inactive U33-5FU55-tRNA, the complex forms in ∼45% yield, largely exceeding that of the modification of the U55-containing substrate (Figure 3a). Further investigations revealed that neither increase of temperature, nor of incubation time significantly increased complex formation (data not shown). The fact that complex formation was instantaneous and occurred at similar efficiency for all three 5FU55-tRNA substrates is clearly incompatible with the interpretation of the complex as a covalent catalytic intermediate, especially given the variegated turnover efficiency of the three substrates. This is most obvious for the 5FU55-U33 substrate, which should not form such a covalent catalytic intermediate in high yield within seconds, since the U33 substrate was not turned over after 1 h.

We conclude that 5FU55-tRNA does indeed form a SDS-stable complex with TruB of *T. maritima*, but that this complex is not the postulated suicide adduct, or of any other relevance to the catalytic mechanism. The fact that we also found the complex not to be stable on a classical urea PAGE (Supplementary Figure S1) further confirms its non-covalent nature. Still, SDS-PAGE, as well as urea PAGE analysis, suggested some differences in the affinity of U55- versus 5FU55-tRNA to TruB (Supplementary Figure S1). Aiming to characterize this affinity, we turned to a spectroscopic characterization of inhibitor tRNA-TruB complex under equilibrium conditions. This approach was also expected to characterize the particular behavior of the inactivating U33-label.

### Spectroscopic changes upon TruB binding

**Figure 4.**
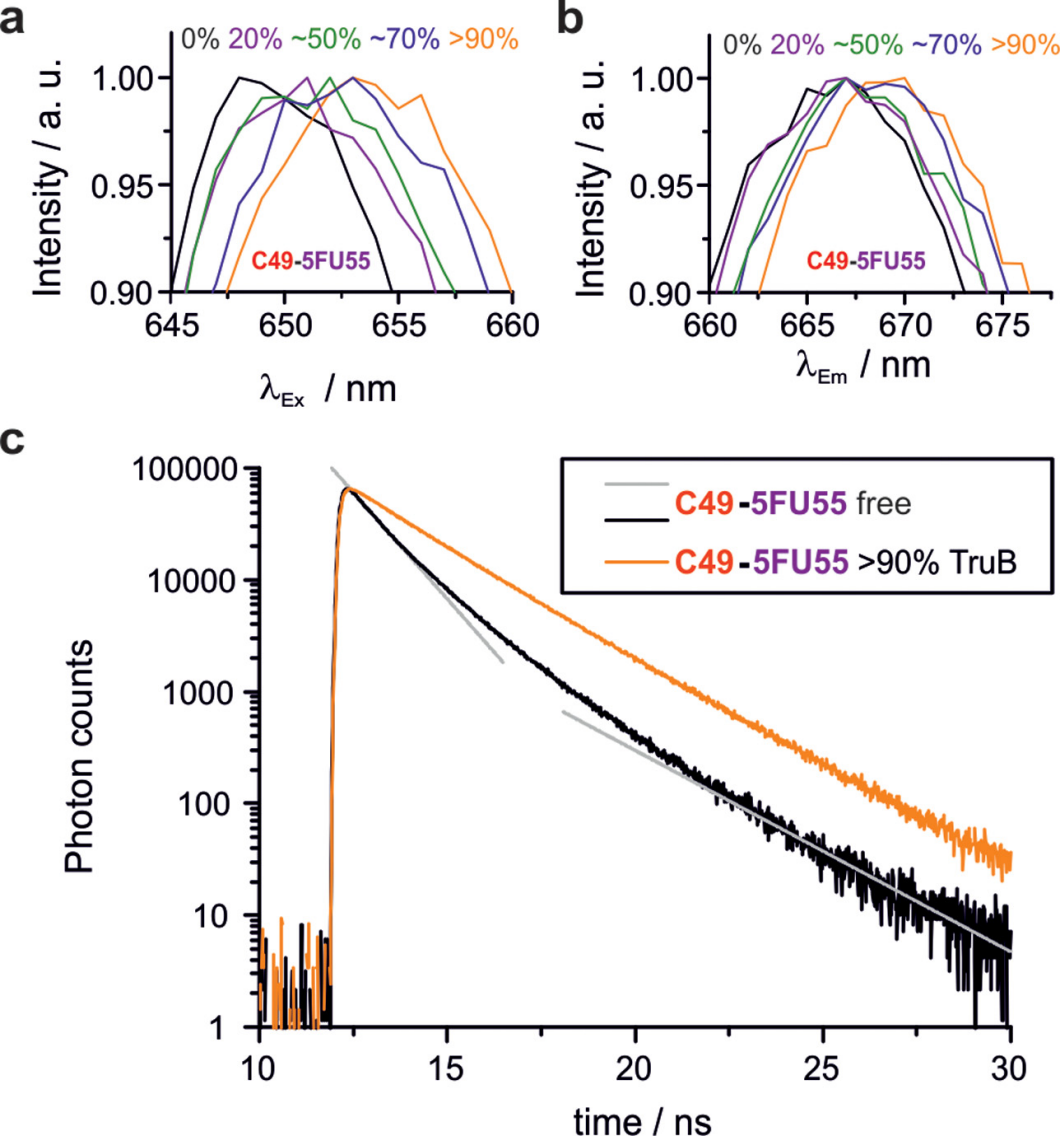
Protein binding (∼% binding is given) causes bathochromic shifts of excitation (**a**, *λ*_Em_ = 668 nm) and emission spectra (**b**, *λ*_Ex_ = 647 nm). Fluorescence decay curves (**c**) differ in absence of TruB and with >90% TruB binding. Gray straight lines indicate bi-exponential fast decay (absence of TruB). The bi-exponential slow decay for >90% TruB binding appears to be mono-exponential due to the log-scale of the *y*-axis.

A commercial spectrometer setup was reconfigured to simultaneously determine time and polarization resolved fluorescence decays, as well as fluorescence excitation and emission spectra at high spectral resolution. Measurements of these properties were performed for both label positions and the U55 and 5FU55 variants, in absence and in the presence of an excess of enzyme, as summarized in Table 1. Remarkably, bathochromic shifts were observed for excitation as well as emission spectra, and these shifts correlated with increasing protein concentrations (see Figure 4 and Supplementary Figure S2), as did polarization resolved fluorescence lifetimes and anisotropy decay (Figure 4, Supplementary Table S1). As is evident from Table 1, the bathochromic shift in excitation was larger than the shift in emission in all cases. Moreover, in the absence of enzyme, both label variants displayed a bi-exponential, fast fluorescence decay with basically identical fluorescence lifetimes. The similarity implies very similar local environment of all fluorescent labels, while the two decay components suggest the existence of two tRNA conformations, of which one provides an environment that is more prone to quenching than the other. This interpretation is in keeping with similar observations of two decay times of *E. coli* tRNA^Phe^ on the single molecule level (61). For all constructs investigated here, the proportions of the bi-exponential fluorescence decay shifted from the fast to the slow decay regime in the presence of enzyme (see Table 1 and Figure 4) suggesting selective stabilization of one conformation by enzyme binding. Remarkably, all shifts in spectroscopic properties were larger for U33-label than for C49-label, with excitation shifting by as much as 8 nm. Anisotropy was also responsive to complex formation and also revealed differences between U33 and C49 that are detailed in the Supporting Information (see Supplementary Table S1, Figure S3 and Supporting Text).

**Table 1.** Changes in spectroscopic properties caused by TruB binding. Values are averages resulting from the experiments given in Figure 5 and at least one endpoint experiment, errors represent the standard deviation.

	Spectra		Composition multi-fit		Lifetimes	
RNA	$\Delta \lambda _{Ex}^{\max } /{\rm nm}$	$\Delta \lambda _{Em}^{\max } /{\rm nm}$	A_1_	A_2_	τ_f1_/ns	τ_f2_/ns
U33-U55	0	0	0.86	0.14	1.12	2.41
+TruB	8.45 ± 0.12	3.46 ± 0.17	0.05	0.95		
U33–5FU55	0	0	0.86	0.14	1.16	2.41
+TruB	8.22 ± 0.44	3.52 ± 0.37	0.14	0.86		
C49-U55	0	0	0.74	0.26	1.12	2.26
+TruB	4.18 ± 0.08	2.50 ± 0.05	0.08	0.92		
C49–5FU55	0	0	0.69	0.31	1.13	2.23
+TruB	4.37 ± 0.08	2.62 ± 0.01	0.13	0.87		

### Spectroscopic titrations

**Figure 5.**
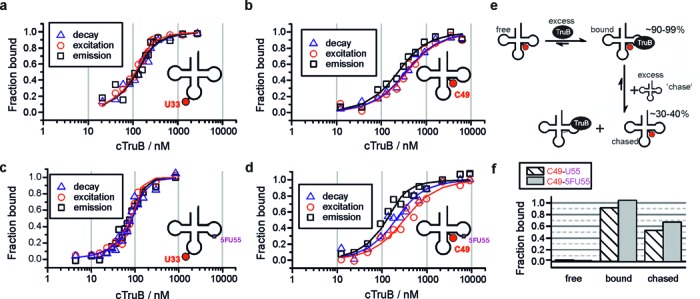
Spectroscopic titration curves of TruB tRNA interaction (*c*_tRNA_ = 200 nM) for U55-tRNAs (**a** and **b**) and 5FU55-tRNAs (**c** and **d**), for all spectroscopic parameters with fits to a 1:1 binding model as solid lines. **(e)** Schematic representation of the chase experiment. **(f)** Chase experiments for C49-tRNA TruB interaction: The fraction bound is averaged over the values obtained from all spectroscopic parameters without TruB (free), with TruB (>90% binding) and after addition of unlabeled U55-tRNA in excess to the complex.

Having identified a suite of spectroscopic parameters as sensitive to TruB binding allowed the determination of the ratio of bound and unbound fractions for a given ratio of tRNA to enzyme. Combining a series of such ratios corresponds to a titration of tRNAs with increasing amounts of recombinant protein. From monitoring the changes upon protein binding upon these parameters in parallel at high spectral resolution, equilibrium dissociation constants were determined. Of note, each parameter showed a slightly different response to the presence of enzyme, and therefore separate different *K*_d_ values were obtained for each parameter. Figure 5a–d show the corresponding titration curves of all four constructs for each of the spectroscopic parameters. Note that lifetime and anisotropy were determined by a global fit assuming a two-component composition of the samples, resulting in a single, combined titration curve. Each curve could be fitted to a one-to-one binding model. As evident from Figure 5, the titration curves of both U55 constructs from the various parameters are all nearly identical. The titration curve sets and therewith the *K*_d_ values of both 5FU55 constructs show somewhat larger deviation among the different parameters. Averaged over all three titration curves, C49-5FU55-tRNA (*K*_d_ = 124.2 nM) binds 2-fold tighter than C49-U55-tRNA (*K*_d_ = 225.8 nM), and at maximum a factor of 4, when comparing affinities resulting from bathochromic emission shifts. On average U33-5FU55-tRNA (*K*_d_ = 3.7 nM) binds somewhat (factor ∼8) more tightly than U55-tRNA (*K*_d_ = 30.7 nM) and at a maximum factor of 14 when comparing affinities resulting from excitation shifts.

This increase of affinity caused by 5FU55 might explain the observation of a complex in SDS-PAGE and urea PAGE, but is clearly too low for a covalently linked catalytic intermediate. To demonstrate reversibility of complex formation by U55 as well as 5FU55 substrates, we performed chase experiments by adding unlabeled U55-tRNA (transcript) to preformed complexes. In this scenario, higher concentration of the unlabeled competitor was meant to drive a fraction of labeled tRNA out of the complex (Figure 5e). Note that 200 nM tRNA was supplemented with 5.23 μM TruB to achieve >95% binding, followed by chase with 5.75 μM competitor concentration. This ∼37× excess reduced binding of labeled tRNA to ∼53% for C49-U55-tRNA and to ∼67% for C49-5FU55-tRNA (Figure 5f). While the absence of a precise *K*_d_ value for the unlabeled substrate precludes exact modeling of this scenario, the higher value for the C49-5FU55-tRNA is in keeping with its higher affinity as determined above and defines an absolute upper threshold for the formation of a hypothetical covalent complex at the difference between the two values, i.e. at 14%.

### Microscale thermophoresis

**Figure 6.**
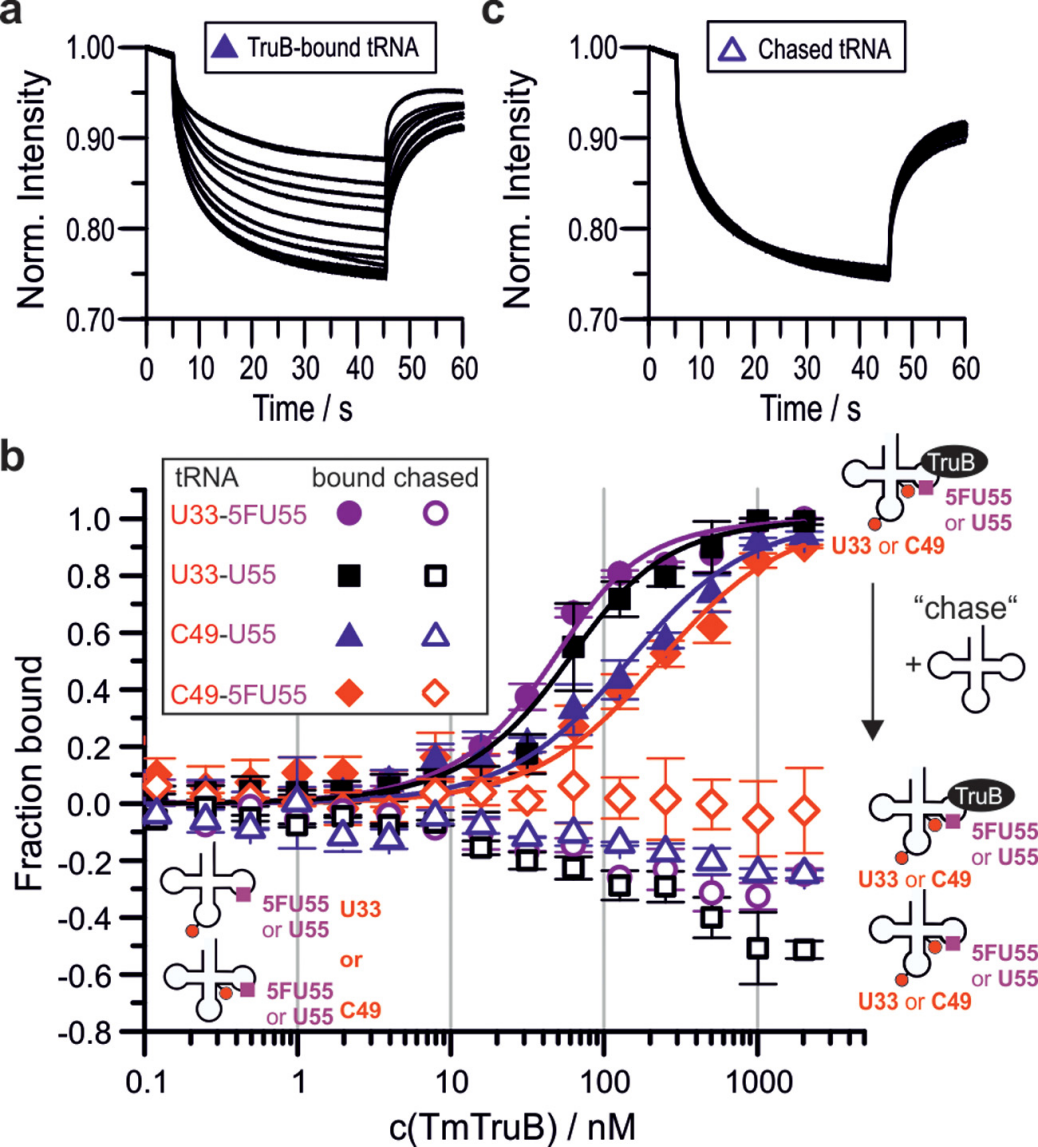
Results of MST experiments. **(a)** Typical set of thermophoresis curves for protein binding. **(b)** Titration curves for *tm*TruB binding (filled symbols) fitted to a one-to-one binding model (solid lines). Empty symbols represent the same samples after addition of unlabeled U55-tRNA in excess (chase). Note that negative values in the chase experiments are presumably caused by the different spectral properties of the bound and the unbound species. Error bars are standard deviations from triplicate measurements. **(c)** Typical thermophoresis curves in chase experiments by addition of unlabeled U55-tRNA.

As a further method for independent verification, we chose MST, an emerging method that has only scarcely been applied to RNA-protein interactions until now, but is highly suitable for working with fluorescently labeled macromolecules (52). Figure 6a shows the primary data of a typical titration experiment using C49-U55-tRNA (see Supplementary Figure S4 for curves of all constructs). Application of MST to the tRNA-TruB interaction (*c*_tRNA_ = 50 nM) produced highly reproducible binding curves (Figure 6b) and corresponding *K*_d_ values in a remarkably short turnaround time. Akin to the fluorescence measurements above, MST measurements are conducted under buffer conditions considered native. In addition, we found that MST facilitates an assessment of the homogeneity of the protein preparation (Supplementary Figure S5) and reproduces *K*_d_ values after 1–3 month intervals with a factor of 2 as maximum deviation (Supplementary Figure S6). Two sets of similar binding curves are apparent, with both U33-tRNAs corresponding to higher affinity (filled squares and circles in Figure 6b) than the C49 derivatives (filled triangles and diamonds).

Here, too, chase experiments were performed to verify that 5FU55-tRNAs could be driven out of the complex by competing tRNA substrate. In comparison to the chase experiments in the cuvette as reported above, the small sample volumes of the MST setup allowed for application of a larger excess of unlabeled competitor and for an assessment at the entire range of protein concentrations used for the *K*_d_ measurements. All labeled tRNAs were used at 35 nM and competed with an ∼100-fold excess (3.3 μM) of unlabeled tRNA. Figure 6c shows the primary data of a chase experiment using C49-U55-tRNA. As is apparent by comparing representative raw data given in Figure 6a and c, the thermophoretic behavior of all labeled tRNAs in the presence of competitor was similar to that of free tRNA. Figure 6b shows that this behavior consistently occurs across a wide range of TruB concentrations (open squares, circles, diamonds and triangles in Figure 6b), clearly illustrating equally reversible complex formation in U55- and 5FU55-tRNAs alike.

**Figure 7.**
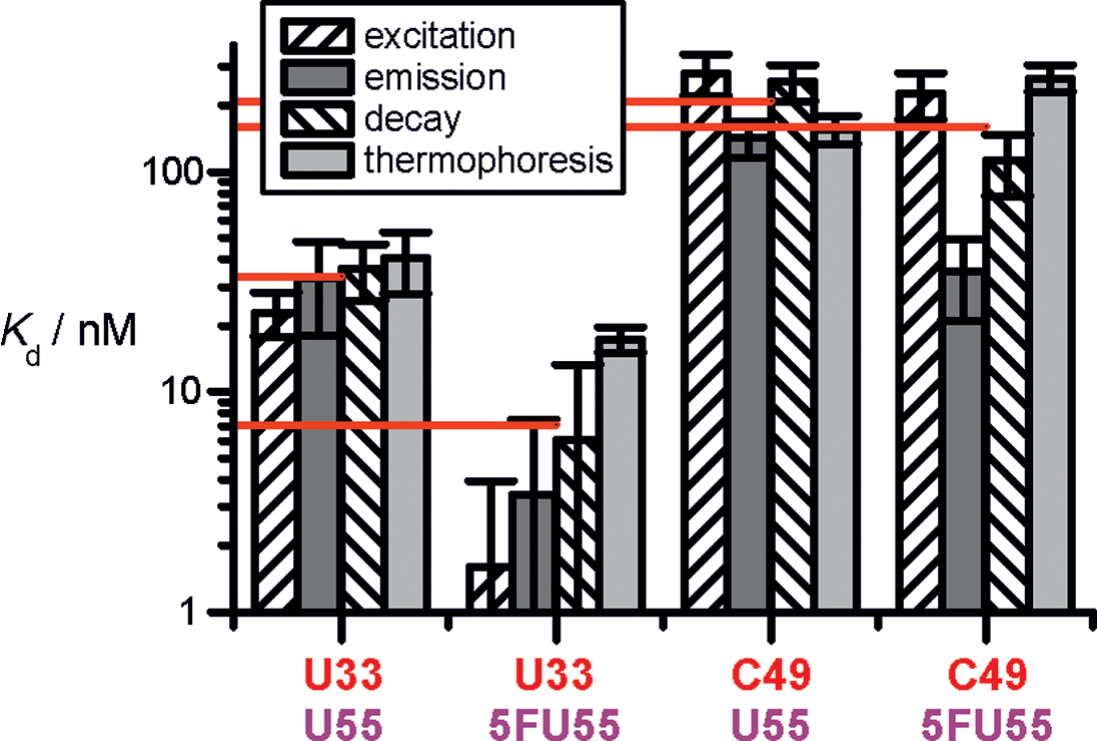
Overview of determined *K*_d_*s*. Error bars represent standard errors derived from fits to the data using Origin 7 software employing the Levenberg–Marquardt algorithm. In case of thermophoresis the standard error results from a simultaneous fit to all three data sets. Red horizontal drop lines indicate arithmetic means over the different *K*_d_ values for each construct.

An overview over all determined affinities is given in Figure 7, with red lines representing averages for each construct. Although *K*_d_ values significantly differ for a given tRNA depending on the method, several important conclusions are substantiated, namely, that (i) U33 binds more strongly than C49 for both U55 and 5FU55 versions and (ii) the introduction of 5FU at the target site 55 slightly increases affinity for the C49, and more notably for U33.

## DISCUSSION

This work connects several novel aspects, first in the investigation of RNA-protein interactions in general, and as a direct consequence insight into the mechanism of a particular RNA modification. Typical approaches to structure–function relationship involve mutation of RNA residues and an assessment of the effect on binding and turnover. In contrast, the use of fluorescent labels on RNA was and still is being met with heightened skepticism, because of its potential for interference with what is considered a physiological recognition event. Here we show, that the use of a dye label in structure–function relationship has multiple advantages over the conventional mutagenesis approach. By attaching fluorescent labels outside and just at the edge of the known recognition site, we generated substrates covering the entire range of inactive to fully active, while the enzyme binding site on the tRNA was left unchanged. A serendipitous discovery was that the label at the remote position U33 almost completely inactivated pseudouridinylation. This is so remarkable, because a TSL minimal substrate (orange in Figure 1) is contained in this molecule, which binds TruB with high affinity. Considering the remote location of the U33 label from the protein binding site, it cannot sterically interfere with binding, nor is the *K*_d_ compatible with a distorted RNA structure at the binding site. Rather, it seems that the catalytic turnover includes a structural rearrangement of the RNA, which is affected by long-range structural crosstalk (59,60) between U33 and the target site. Such a rearrangement is known from the RNA modification enzyme archeosine tRNA-guanine transglycosylase (62), where it involves a substantial induced fit to a so-called λ-tRNA. There is circumstantial evidence that TruB has to undo tertiary interactions to access the target site (10). Interestingly, our and previously published (61) fluorescence lifetime data suggest two alternative tRNA conformations, one of which is apparently stabilized by TruB binding. This conformation might be similar to the λ-tRNA and possibly important for catalysis, although the data on the U33 labeled tRNA, which is inactive but shows both conformations, suggest that at least one more conformation might be required for pseudouridine formation. Unfortunately, the available crystal structure is not informative in this respect, since it includes the TSL minimal substrate rather than the full tRNA (23,25,27).

The particularity of the U33 label also became evident in spectroscopic characterization, which revealed a ∼8 nm bathochromic shift of the excitation maximum, about twice as much as for the C49 label, and points to substantial differences in the TruB interaction with the respective RNAs. The actual cause for the observed changes of the dyes spectroscopic properties is not easily identified due to a range of complex effects, including the dye's local viscosity (63) and changes in the cis-isomerization rate of cyanine dyes (64–68). Reportedly, dye-protein interactions cause bathochromic shifts of excitation and emission in spectra of Cy5 (65) and can, similar to dye-nucleic acid interactions, increase the dye's fluorescence lifetime by decreasing the rate of cis-isomerization (63,65). The magnitude and tendency of bathochromic shifts, as well as the increase in fluorescence lifetime we observe for TruB binding (see Table 1) correspond well to a previous study on binding of biotinylated Cy5 to the protein streptavidin (69).

While further investigations must define the differential effects of the labels in more detail, the benefit of using differential dye labels *en lieu* of classical mutagenesis becomes evident in the possibility to determine *K*_d_ values from multiple parameters and by multiple fluorescence-based approaches. While the determination of *K*_d_ values via fluorescence anisotropy is routine (70–73) and fluorescence lifetime or intensity increase were used in rare cases (74,75), the power of the combined application of all four spectroscopic parameters to the investigation of bimolecular binding events has, so far, gone unnoticed (73–78).

As Figure 7 clearly shows, *K*_d_ values can diverge by a factor of 11 at worst (U33–5FU55), while the others are in keeping with generally accepted error ranges in the biophysical characterization of RNA-protein complexes. Still, the ensemble of measurements confers confidence that binding of 5FU55-tRNA is only moderately stronger than that of the U55 substrates. It should be noted that high affinity for a weak substrate as U33 labeled tRNA is not uncommon for Ψ synthases (58) and reflects the low binding specificity of these enzymes.

The three substrates investigated here (Figure 3) display variegated enzymatic turnover on a >1 h time scale, yet their 5FU55 containing counterparts all form complexes with TruB within minutes, which yield clearly visible bands on SDS-PAGE in ∼40% yield. The characteristics of these bands, in particular that of the inactive U33 variant, is in absolute conflict with their previous interpretation as a covalent suicide intermediate in the catalytic cycle of pseudouridine formation (23). Rapid formation of a covalent complex formation following 1 min incubation (Figure 3), is not compatible with the complex resulting from catalytic action of an enzyme as slow as TruB (6). Furthermore, a covalent complex should be observable on both, SDS and Urea PAGE, which is the case for the distantly related Ψ synthase TruA (8), but not for TruB (Figure 3 versus Supplementary Figure S1). Finally, performed chase experiments analyzed by fluorescence emission and by MST, failed to give any indication of a covalent intermediate.

Of note, covalent suicide intermediates of 5-fluoropyrimidines have been investigated in a number of reaction mechanisms (21,34,40–41,79) bearing significant, albeit incomplete, similarity to what is known of pseudouridine formation. Some intermediates are stable in urea (41,44) and all are stable during heating (35–37,39–40). The fact that a suicide adduct was postulated for Pus enzymes as well, is likely related to the fact that all crystal structures of 5FU-containing substrate in complex with Pus enzymes contained a clearly identified, partially turned-over 5FU derivative (23,25–26). These, however, did not contain a covalent linkage between RNA and protein, and were assumed to result from slow hydrolysis of the latter during crystallization. In contradiction to this hypothesis Stroud *et al.* recently published a ‘fortuitous’ adduct of RIuB, which contained a partially turned-over 5FU derivative (31) covalently linked via a non-essential tyrosine (26). Since this tyrosine is not a general feature of Pus enzymes, the covalent tyrosine adduct cannot be of general importance to this discussion. On the other hand, this example shows that the covalent linkage is stable enough to survive crystallization conditions.

We conclude that the observed bands in SDS-PAGE correspond to an RNA-protein complex that is resistant to SDS, but that cannot be of covalent nature. Stable protein–protein complexes (80–83) and even catalytic activity have indeed been observed in the presence of SDS (84,85). Fluorine substitution was reported to increase binding affinity in numerous complexes of proteins with small molecules, and both, extra hydrogen bonding and an increase in hydrophobicity, have been discussed (86). In the case at hand, however, SDS resistance is due to the presence of 5FU55, but does not solely originate from the higher affinity conferred by 5FU55: Although the *K*_d_ of U33-U55-tRNA is lower than that of C49–5FU55-tRNA, only the latter tRNA forms a SDS-stable complex.

We propose that 5FU containing RNAs may act as mere competitive inhibitors of Pus enzymes in general, without forming stable covalent intermediates. This is in line with our previous studies of Pus1, which failed to convert 5FU on a biochemically relevant time scale, but did show complex dissociation on urea PAGE that was weaker for 5FU- than for U-tRNA, implying that 5FU-RNA forms a non-covalent complex more resistant to denaturants (87). For several Pus enzymes of *E. coli* the Mueller group observed turnover of 5FU-RNA (8,32), although not all of them formed SDS-stable complexes (32). These results seem to be contradictory if the SDS-stable complex band was to result from a catalytic suicide adduct that could be heat disrupted into the generally observed product of 5FU-RNA turnover. Particularly remarkable seemed the difference in SDS-stable complex formation by two TruB variants: The *T. maritima* variant does (23) (and *vide supra*) and the *E. coli* enzyme does not (32) form SDS-stable complexes with 5FU-RNA. As our results show, SDS-stable complex formation is independent of catalytic turnover, thereby allowing the above described discrepancies.

## CONCLUSION

We have presented a combination of *K*_d_ measurements with chase experiments, and a contradiction of SDS-resistant 5FU55-tRNA-TruB complex yields with catalytic turnover data. These experiments clearly show that the interpretation of the ominous band in protein gels as covalent intermediate in the catalytic formation of pseudouridine by TruB cannot be upheld any more. This is of significant impact to the community, as it represented the only tangible evidence (23) for this intermediate. Importantly, 5FU has enjoyed widespread use as an alleged suicide inhibitor because it supposedly led to covalently linked RNA-Pus complexes in biochemical and biophysical studies (23,25,28,88–89). Importantly, these findings do not resolve the longstanding discussion about the mechanism of pseudouridine formation. Of the three mechanisms proposed, namely, acylal, glycal and Michael addition mechanism (discussed in the Supporting Information and shown in Supplementary Figures S10 and S11), the experimental basis for the latter two remains untouched, while a limited amount of experimental support for the Michael-addition mechanism might erode if carefully reinterpreted. Of note, the above conclusion could only be reached for two reasons, namely, (i) the use of fluorescent dyes rather than conventional mutagenesis in structure–function studies and (ii) a suite of fluorescence-based measurements of RNA-protein interactions, including, in particular, the exploitation of bathochromic shifts and MST.

## SUPPLEMENTARY DATA

Supplementary Data are available at NAR Online.

SUPPLEMENTARY DATA
