# Supplementary material for: Dye label interference with RNA modification reveals 5-fluorouridine as non-covalent inhibitor
Source: Nucleic Acids Res. 2014 Oct 9;42(20):12735–45. doi: 10.1093/nar/gku908 (PMC4227767; doi:10.1093/nar/gku908)
Supplement: SUPPLEMENTARY DATA [file supp_42_20_12735__index.html]

Dye label interference with RNA modification reveals 5-fluorouridine as non-covalent inhibitor — Dye label interference with RNA modification reveals 5-fluorouridine as non-covalent inhibitor — SUPPLEMENTARY DATA 

# Dye label interference with RNA modification reveals 5-fluorouridine as non-covalent inhibitor

## SUPPLEMENTARY DATA

**Files in this Data Supplement:**

- SUPPLEMENTARY DATA
