# Supplementary material for: Dye label interference with RNA modification reveals 5-fluorouridine as non-covalent inhibitor
Source: Nucleic Acids Res. 2014 Oct 9;42(20):12735–45. doi: 10.1093/nar/gku908 (PMC4227767; doi:10.1093/nar/gku908)
Supplement: SUPPLEMENTARY DATA [file supp_gku908_nar-01721-r-2014-File009.doc]

Supporting Information - Dye label interference with RNA modification reveals 5-fluorouridine as non-covalent inhibitor

Felix Spenkuch^1^, Gerald Hinze^2^, Stefanie Kellner^1^, Christoph Kreutz**^3^**, Ronald Micura^3^, Thomas Basché^2^ and Mark Helm^1^,*

^1^Institute of Pharmacy and Biochemistry, University of Mainz, Staudingerweg 5, D-55128 Mainz, Germany

^2^Institute of Physical Chemistry University, of Mainz, Duesbergweg 10-14, D-55128 Mainz, Germany

^3^Institute of Organic Chemistry, Center for Molecular Biosciences (CMBI), University of Innsbruck, Innrain 80/82, A-60230 Innsbruck, Austria

* To whom correspondence should be addressed. Tel: +49 6131 39 25731; Fax: +49 6131 39 20373; Email: mhelm@uni-mainz.de

Present Address: Stefanie Kellner, Department of Biological Engineering, Massachusetts Institute of Technology, 56-787b, 77 Massachusetts Ave., Cambridge, Massachusetts, 02139, USA

Contents:

C49-tRNA-TruB interaction probed by urea PAGE 2

**Figure S1.** tRNA-TruB interaction probed by urea PAGE. 2

Spectra of U33-tRNAs resulting from TruB titration 3

**Figure S2.** Representative spectra of U33-tRNA 3

Effects of label position and TruB binding on anisotropy decay 3

Table S1. Changes in Anisotropy upon TruB binding. 3

**Figure S3.** Detail of the tRNA-TruB docking model 4

Additional MST Experiments 5

**Figure S4.** Representative MST sets of all constructs used 5

**Figure S5.** Influence of protein inhomogeneity on MST 5

**Figure S6.** Longterm reproducibility of MST. 6

Oligonucleotides 6

**Figure S7.** ESI-MS of the 5-fluorouridine-containing 25-mer oligonucleotide. 6

Table S2. Oligonucleotides used for splinted ligations. 6

LC-MS measurements of pseudouridine formation 7

Table S3. Raw data from LC-MS/MS measurements. 8

**Figure S8.** Measurements performed by LC-MS/MS 9

On the possible mechanisms and mechanistisc steps of pseudouridine formation by TruB

**Figure S9**. Pseudo-tertiary structure of yeast tRNA^Phe^ and contacts of T. maritima TruB 10
to the TSL minimal substrate.

**Figure S10.** Possible mechanisms of pseudouridine formation 11

**Figure S11.** The assumed product of turnover by pseudouridine synthases and the various 11
final products for either 5FU or U turnover

**Figure S12.** The glycal mechanism for pseudouridine formation as proposed by Miracco and Mueller 13

Supplementary references 14

C49-tRNA-TruB interaction probed by urea PAGE

Prior to enzyme addition C49 tRNA was folded by heating for 4 min at 75 °C in water and cooling to room temperature in 15 min after addition of MST buffer. All reactions were incubated in 10 µl total volume 2 µM tRNA and TruB concentrations for 10 min at 25 °C, following 5 min incubation in 0.5 x the respective loading dye (SDS buffer or 90% formamide in TBE (FA)) at either 25°C (Heat -) or 95 °C (Heat +). A 20x30 cm 10% 8 M Urea PAGE was run at 100 V and room temperature. Post run the gel was scanned with a GE Healthcare Typhoon 9400 for Cy5 (excitation 633 nm, emission BP670). As evident from a Coomasie stain, TruB incubated in formamide only barely enters the gel as a diffuse streak that runs on similar height as TruB in SDS buffer (data not shown). The Cy5 scan of the same gel is given in Figure S2 and allows the following observations:

(i) very weak autofluorescence of TruB can be detected, if SDS buffer is used (lanes 1, 10, 11, 18 and 19)

(ii) no complex formation with U55-tRNA occurs (lanes 10-13)

(iii) no complex for 5FU55-tRNA, if the reaction is heated to 95 °C prior to gel loading (lanes 1+3).

(iv) as with SDS PAGE, the complex is 5FU55-specific, but detectable in only ~1% yield in SDS loading buffer (lane 2), a stark contrast to the ~40% detectable complex on SDS PAGE (Figure 3 in the main text)

(v) In formamide buffer dissociation of the 5FU55-tRNA TruB complex is evident from heavy smearing (lane 4). In summary the ‘stable’ complex is neither resistant to urea, nor to formamide and therewith an SDS-related artifact.


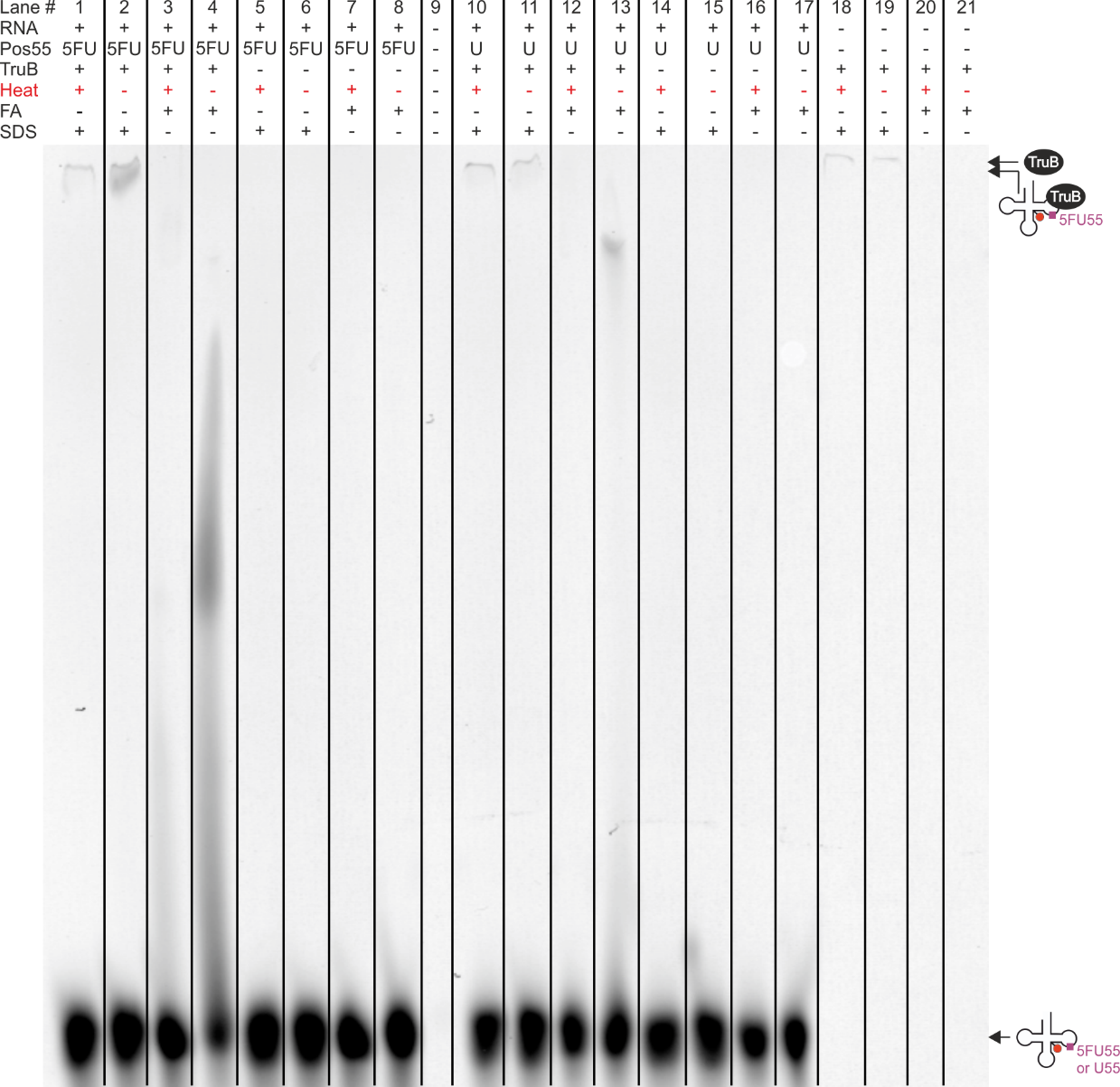


**Figure S1.** TruB-C49-tRNA interaction assessed by 8 M Urea PAGE. Four conditions were tested: Prior to gel loading the complex reaction was either incubated for 5 min in either formamide or SDS buffer as loading dye at 25 °C or 95 °C. Detection by Cy5 scan (excitation 633 nm, emission 670BP30).

Spectra of U33-tRNAs resulting from TruB titration


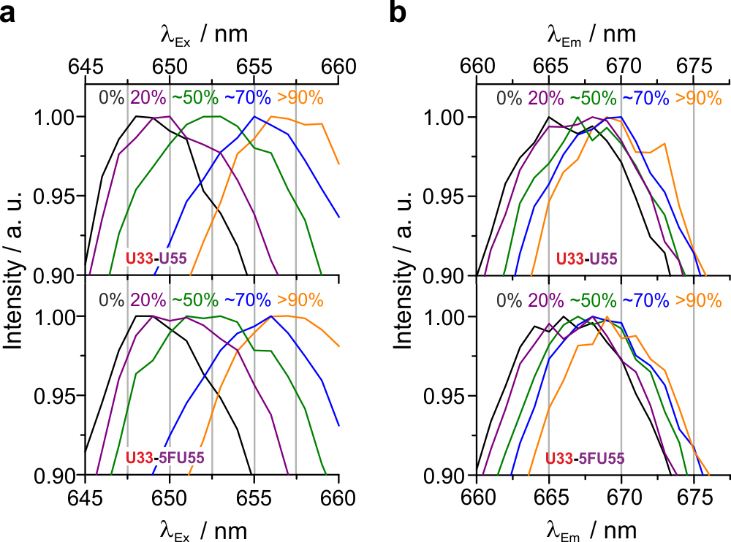


**Figure S2.** Representative spectra of U33-tRNA as resulting from TruB titration. Protein binding (~% binding is given) causes bathochromic shifts of excitation (**a**, U55 above, 5FU55 below,
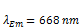
) and emission spectra (**b**, U55 above, 5FU55 below,
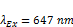
).

Effects of label position and TruB binding on anisotropy decay

Since the sample contains populations that differ in fluorescence life time, as well as rotational correlation times, time resolved fluorescence data has to be treated as so‑called associated anisotropy decay to allow for deconvolution of anisotropy decay and fluorescence lifetimes (1). Since systems with more than two correlation times are difficult to address (1) and could therefore let us interpret too much into our data, we chose a simple two component system, where components differ from each other in both, fluorescence lifetime and correlation time. The parallel and the perpendicular component are described by the following equations:


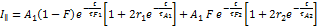
 Eq.1


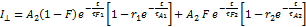
 Eq.2

Here (1-F) and F describe the fractional abundance of the two components, A1 and A2 are scaling amplitudes for both data sets
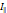
 and
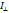
, τ_F1_ and τ_F2_ the fluorescence decay times, anisotropy amplitudes are given by r_1_ and r_2_ corresponding to rotational correlation times τ_A1_ and τ_A2_, respectively. Anisotropy values for all constructs are given in Table S2. One component shows a fast anisotropic rotation, the other a rotational correlation time typical for a dye trapped in a protein complex.^1^

The rotational correlation times are remarkably similar for all constructs with a slightly faster τ_A1_ for U33-tRNA. The amplitudes differ not for 5FU55 *vs.* U55, but for the dye label position: For U33-tRNA the amplitudes of both components are similar, while the slow rotating component is nearly negligible for C49-tRNA.

Table S1. Changes in Anisotropy upon TruB binding.

| RNA | τ_A1_ / ns | τ_A2_ / ns | r_1_ | r_2_ |
| --- | --- | --- | --- | --- |
| U33-U55 | 0.56 | 22.4 | 0.23 | 0.11 |
| U33-5FU | 0.66 | 21.1 | 0.22 | 0.14 |
| C49-U55 | 0.52 | 31.7 | 0.21 | 0.05 |
| C49-5FU | 0.53 | 20.2 | 0.20 | 0.03 |

The tRNA-TruB docking model (2) might explain at least one half of the observations: As is shown in Figure S3, C49 (depicted as a red structure) lies ‘on top’ of the enzyme shown as grey surface. The red inset in Figure S3 shows that the dye is attached to the ‘C-H edge’ of C49, positioning it towards the RNA backbone. The position marked with a blue arrow corresponds to the circle in the crystal structure, thereby depicting the dye attachment site.

Considering the long linker attaching the dye to the base, one could imagine a quite undisturbed rotation of the dye ‘above’ the tRNA-TruB complex. Consistent with this hypothesis, we propose that r_2_ of C49-tRNA is low, because the dye is still highly mobile in the complex and the resulting fast (almost isotropic) rotation cancels out the slow component. In reverse a larger r_2_ for U33-tRNA implies an effective low mobility of the enzyme-bound U33-label, presumably due to long distance intramolecular rearrangement, as discussed in the main text.


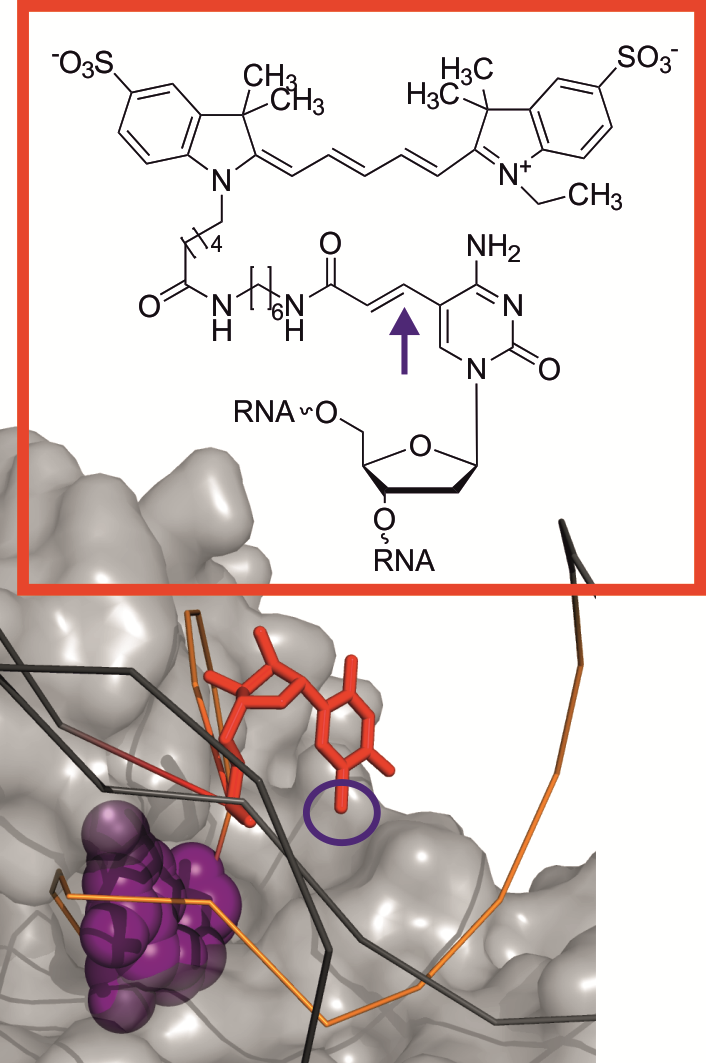


**Figure S3:** Detail of tRNA-TruB docking model (2): TruB gray surface, tRNA represented as black ribbon, minimal substrate in orange and substrate position 55 as purple sphere. The red structure shows m^5^C49 native to yeast tRNA^Phe^ with the methyl group at position 5 marked by a dark-blue circle. The structure of Cy5 attached to the base dC is shown as inset. A blue arrow marks the position corresponding to the blue circle, thereby depicting the dye attachment site.

Additional MST Experiments:

Only a fresh, gel filtrated protein preparation generated clear results, as shown in Figure S4.


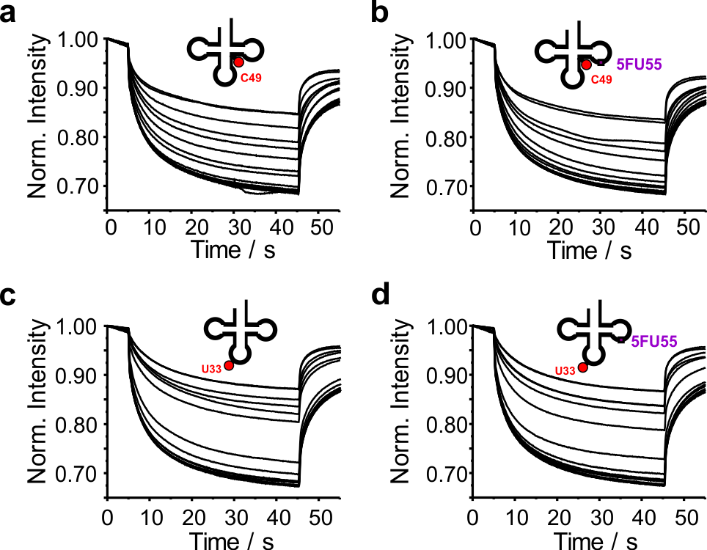


**Figure S4.** Representative MST sets of all constructs used: **a)** C49 U55,**b)** C49 5FU55, **c)** U33 U55, **d)** U33 5FU55.

In contrast thermophoresis curves of an old protein stock showed heavy fluctuations (Figure S5 a) resulting in a titration curve lacking proper fits with either a Hill model, or a one-to-one binding model (Figure S5 b). Both models yielded similar, low affinites: EC50 = 1592 nM for the Hill model and K_d_ = 1520 nM for one-to-one binding. Thorough centrifugation results in smoother traces (Fig.S5 c).


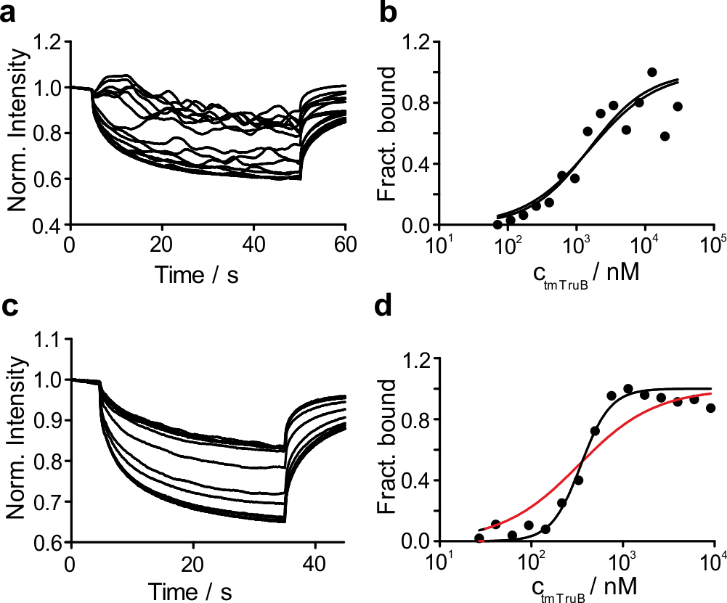


**Figure S5.** Influence of protein inhomogeneity on MST experiments. **a)** Thermophoresis curves of 5 year old 300 µM protein. **b)** Titration curve resulting from a) showing low affinities from one-to-one binding and Hill model (solid black lines). **c)** Thermophoresis curves of centrifuged protein. **d)** Titration curve and fits resulting from c): One-to-one binding model (red line) and Hill model (black line).

However the resulting steep titration curve could not be fitted to a one-to-one binding model (red line in Figure S5 d). A Hill fit (black line in Figure S5 d), yielded n = 2.68 and EC50 347.8 nM, more than 10x above the actual affinity.

Affinity of *tm*TruB for the U33-5FU55 construct was measured three times over 3 month with variations up to a factor of 2 and a precision value of 52%, as apparent from Figure S6.


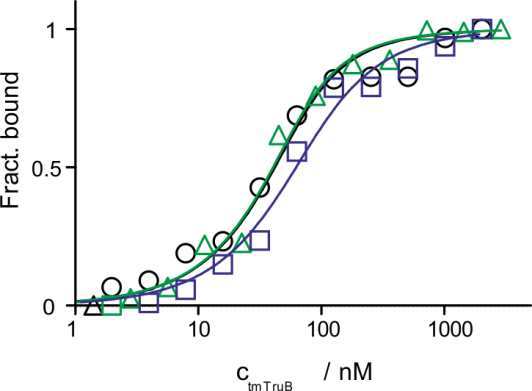


**Figure S6.** Longterm reproducibility of MST. Triplicate of a TruB-U33-5FU55 titration, while each run was 1 month apart from the other. K_d_ values as resulting from the fit to a one-to-one binding model (solid line): empty squares: 37.31 nM; empty triangles: 15.49 nM; empty circles: 16.89 nM.

**Oligonucleotides**


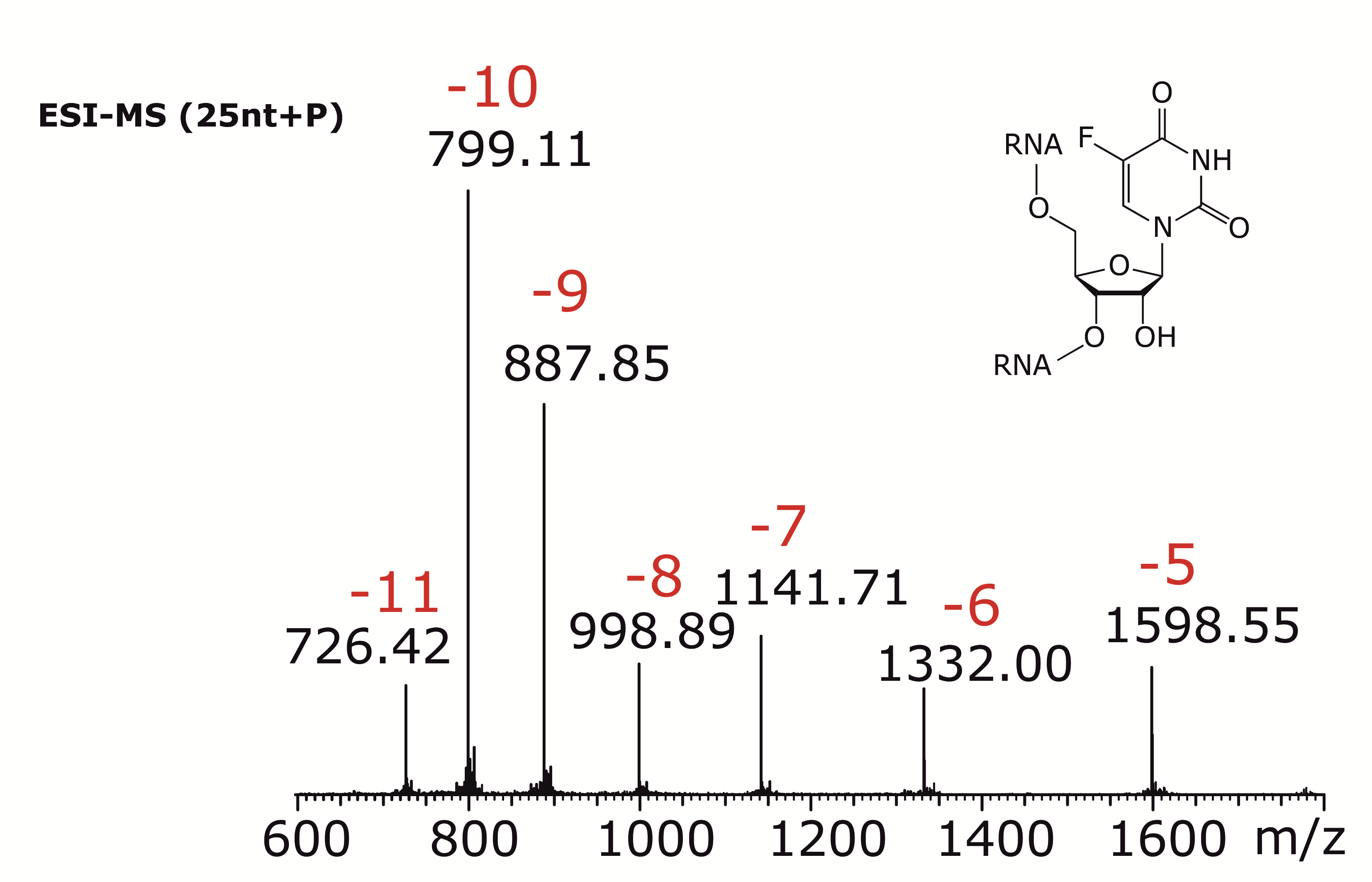


**Figure S7:** ESI-MS of the 5-fluorouridine-containing 25-mer oligonucleotide. Peaks for the five-times to ten-times charged molecular ions are shown, while z–values are annotated in red and m-values in black. Calculated mass of once-charged ion is 7999.76, which correlates well with the various multiple charged peaks visible.

Table S2. Oligonucleotides

| type | fragment | Sequence 5‘ => 3‘ | modification |
| --- | --- | --- | --- |
| RNA | 5' | GCG GAU UUA GCU CAG UUG GGA GAG CGC C |  |
| RNA | middle | pAGA CUG AAG AUC UGG AGG UCC UG |  |
| RNA | middle | AGA C**U***G AAG AUC UGG AGG UCC UG | **U***: Cy5 U33 |
| RNA | middle | AGA CUG AAG AUC UGG AGG UC**C*** UG | **C***:Cy5 C49 |
| RNA | 3‘ | UGU UCG AUC CAC AGA AUU CGC ACC A | None: U55 |
| RNA | 3‘ | pUGU **U**CG AUC CAC AGA AUU CGC ACCA | **U**: 5FU55 |
| DNA | splint | TGG TGC GAA TTC TGT GGA TCG AAC ACA GGA CCT CCA GAT CTT CAG TCT GGC GCT CTC CCA ACT GAG CTA AAT CCG C | |

LC-MS measurements of pseudouridine formation

1. Turnover assay

Prior to enzyme addition tRNA was folded by heating for 4 min at 75 °C in water and cooling to room temperature in 15 min after addition of MST buffer. Following enzyme addition all samples were incubated in 1x MST at 80 °C for 70 min to facilitate enzymatic turnover.

2. LC-MS sample preparation

Subsequently pseudouridine (Ψ) formation was quantified by LC-MS as described below. The tRNA transcript and U33, C49 constructs (final concentration 0.5-1.5 pmol/µL) were boiled for 10 minutes at 95 °C and subsequently dissolved in 20 mM NH4OAc pH 5.3. The samples were incubated for 2 h at 70 °C in the presence of 0.0003 U nuclease P1 (Roche Diagnostics, Mannheim, Germany) per 10 pmol RNA, which leads to a complete degradation to mononucleotides. Snake venom phosphodiesterase (Worthington, Lakewood, USA) was then added to a concentration of 0.06 U per 100 µg RNA, and the mixture was incubated at 37 °C for another 1 h. Finally to convert the resulting mixture of mononucleotides to free nucleosides, 1/10 vol of 10x FastAP buffer (Fermentas, St. Leon-Roth, Germany) was added, followed by 3/20 vol of H_2_O, and 1 U of FastAP Thermosensitive Alkaline Phosphatase (FastAP stock at 1 U/µL; from Fermentas, St. Leon-Roth, Germany). The mixture was incubated for 1 h at 37 °C. An internal standard containing ^13^C labeled pseudouridine was added (10 Vol% of sample volume) to the samples for absolute quantification of pseudouridine turnover. This internal standard was received by digestion of total tRNA from *E. coli* grown in a M9 media with ^13^C-glucose as the only carbon source. For calibration measurements, commercially available guanosine (Sigma-Aldrich, Munich, Germany) and pseudouridine (Berry & Associates, Dexter, USA) were used.

3.0 LC-MS and LC-MS/MS analysis

The digested tRNA was analyzed on an Agilent 1260 series equipped with a diode array detector (DAD) and Triple Quadrupol mass spectrometer Agilent 6460. A Synergy Fusion RP column (4 µm particle size, 80 Å pore size, 250 mm length, 2 mm inner diameter) from Phenomenex (Aschaffenburg, Germany) was used at 35 °C. The solvents consisted of 5 mM ammonium acetate buffer adjusted to pH 5.3 using acetic acid (solvent A) and pure acetonitrile (solvent B). The elution started with 100% solvent A followed by a linear gradient to 8% solvent B at 10 min and 40% solvent B after 20 min. Initial conditions were regenerated by rinsing with 100% solvent A for 10 minutes. The flow rate was 0.35 mL/min.

The effluent from the column was first measured photometrical at 254 nm by the DAD followed by the mass spectrometer equipped with an electrospray ion source (Agilent Jet Stream). ESI parameters were as follows: gas temperature 300 °C, Gas flow 5 L/min, Nebulizer pressure 35 psi, Sheath gas temperature 350 °C, Sheath gas flow 12 L/min, capillary voltage 3500 V. The MS was operated in positive ion mode to monitor selectively the transitions for pseudouridine (m/z: 245🡪209) and ^13^C-pseudouridine (m/z: 254 🡪 218) in dynamic SRM mode.

4.0 Calculations for quantification of pseudouridine in tRNA

All samples were measured as described above. The data was then processed using the UV data for quantification of injected tRNA and MS data for quantification of pseudouridine.

4.1. Quantification of injected tRNA

For each sample, the UV_254 nm_ area of the main nucleoside guanosine was used to calculate the amount of injected tRNA (23 guanosine residues per tRNA). Here, calibration measurements of guanosine dilutions were applied for exact quantification (Figure S6 a) left).

4.2. Quantification of pseudouridine turnover

For both pseudouridine and its ^13^C-labeled derivative, the MS/MS peaks were integrated. The peaks were correlated by a previously determined response factor (r_f_) (Figure S6 a) right), which allows the calculation of the total amount of pseudouridine per sample (shown below for incubation of tRNA transcript with TruB). All other samples were analyzed, accordingly.

For calculation of the amount of injected pseudouridine (Ψ), the following equation is used


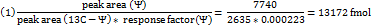


The amount of injected pseudouridine is correlated to the amount of injected RNA. This amount is received by analysis of the guanosine peak in the 254 nm chromatogram. The peak area for this measurement is 800. This signal contains the signal from the tRNA guanosine and the ^13^C guanosine signal from the internal standard (ISTD). The signal intensity for the added internal standard is 129.82. Therefore, the share of guanosine signal arising from the tRNA is


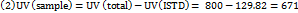


Now, the amount of injected guanosine in pmol can be calculated by using a guanosine calibration factor (2.21).


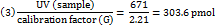


To assess the amount of RNA, the number of guanosines of the analyzed tRNA must be known. Here, 23 guanosine nucleosides are contained in the sequence.


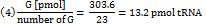


Finally, the amount of pseudouridine (Ψ) can be correlated with the amount of tRNA:


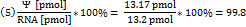


Table S3: Raw data from LC-MS/MS measurements.

| RNA | Precursor Ion | Product Ion | Retention time | Peak area |
| --- | --- | --- | --- | --- |
| pseudouridine ^13^C | 254 | 218 | 3.661 | 2635 |
| pseudouridine | 245 | 209 | 3.671 | 7740 |


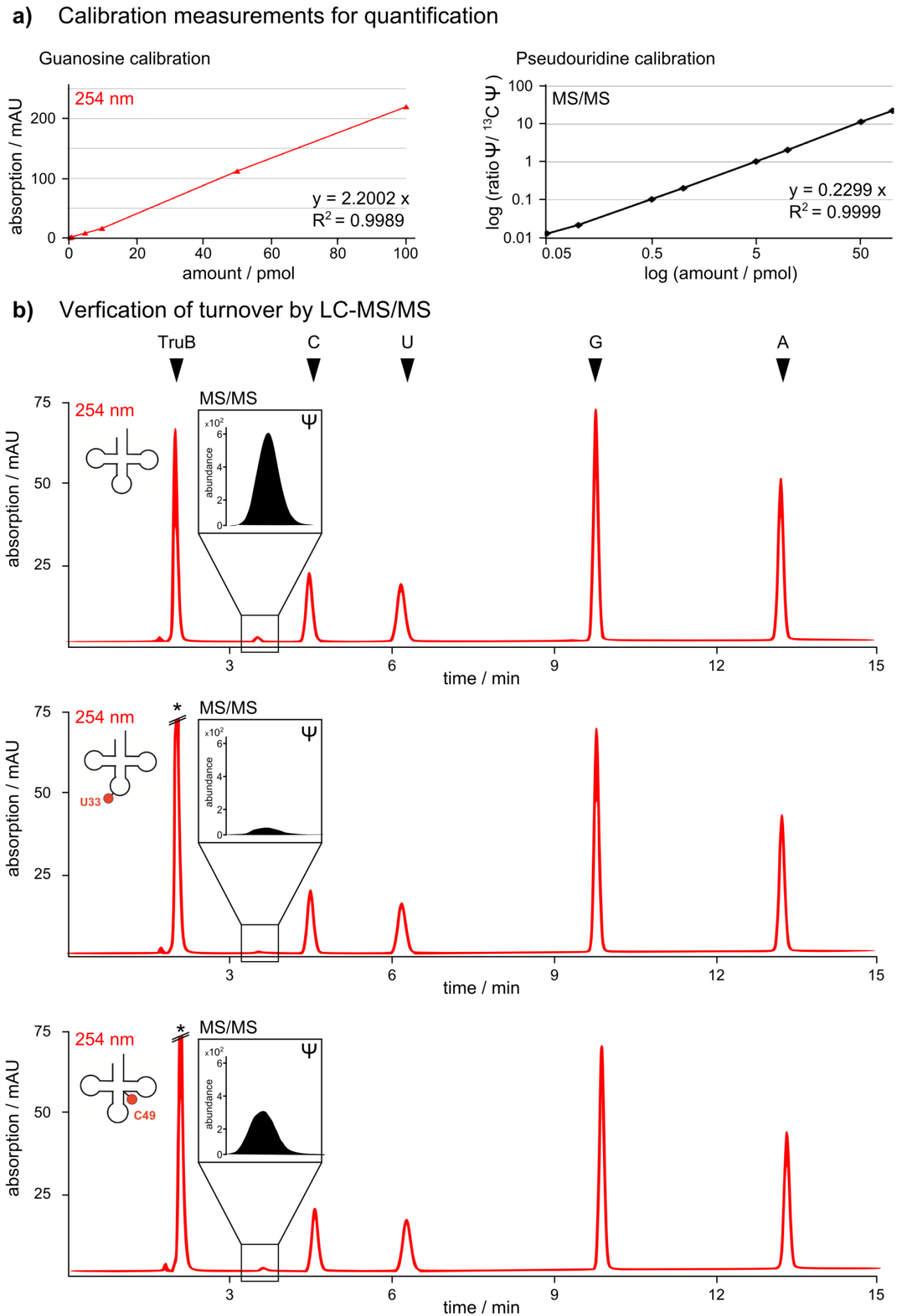
**Figure S8:** Measurements performed by LC-MS/MS analysis for verification of turnover efficiency. a) calibration curves of guanosine by UV detection and pseudouridine by MS/MS detection. Note: The MS/MS plot of pseudouridine already includes the adjustment of detection efficiency by usage of ^13^C labeled pseudouridine as an internal standard. b) UV chromatograms (red) and MS signals of pseudouridine (black) of TruB incubated transcript, U33-U55 construct and C49-U55 construct. No pseudouridine could be detected in the negative controls (TruB without RNA and transcript without TruB, respectively).

**On the possible mechanisms and mechanistisc steps of pseudouridine formation by TruB**

Members of the TruB family of pseudouridine synthases modify U55 in the T stem loop of tRNA (blue in Figure S9, substrate position is shown in bold). Judging from cocrystal structures with the TSL minimal substrate (4, 5), the enzyme forms various contacts to the TSL (contacts are shown red in Figure S9 b). Tertiary interactions that may get disrupted by TruB binding to full length tRNA are shown in red in Figure S9 a.


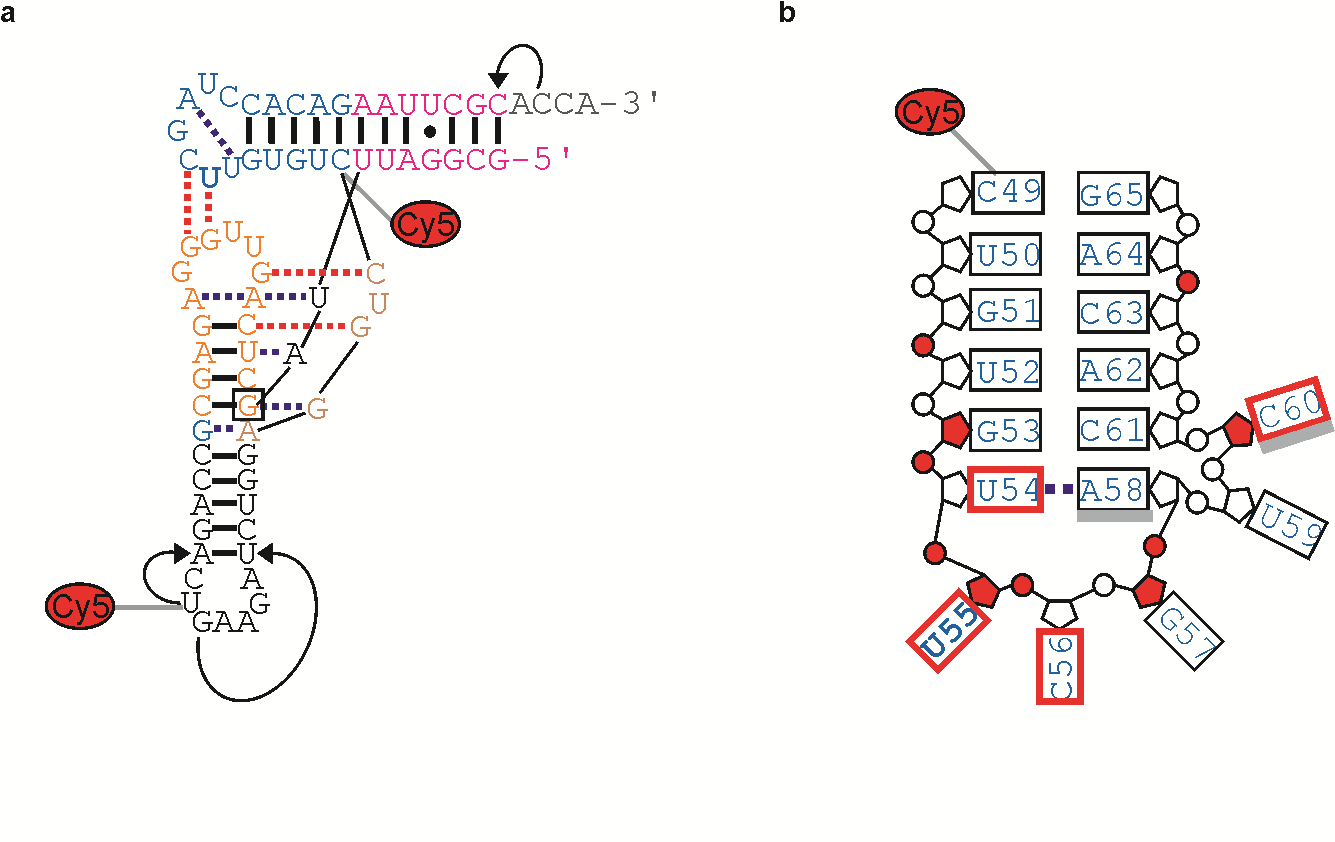


Figure S9. Pseudo-tertiary structure of yeast tRNA^Phe^ and contacts of T. maritima TruB to the TSL minimal substrate. a Tertiary interactions forming the canonical L-shape are depicted by dashed lines, arrows indicate base stacking (3). Canonical tRNA domains are color coded: The Anticodon stem is depicted in purple, the D stem loop is shown in orange, the anticodon stem loop in black, the variable loop in brown and the T stem loop is depicted in blue with the substrate position U55 shown in bold letters. b The T stem loop minimal substrate in TruB-bound conformation as found in the cocrystal (4). Site that are in contact with *Thermotoga maritima* TruB are depicted in red, amino acid stacking to A58 and C60 is indicated by a gray bar.

In the current model of TruB catalysis, established from structures of the apo-enzyme (4, 5) and cocrystals (2, 4-5), as well as pre-steady state kinetics (6, 7), TruB catalysis on tRNA has to comprise of at least four steps:

1. Initial tRNA binding
2. Conformational changes in enzyme and RNA, including flipping of bases out of the T loop into the catalytic center of the enzyme
3. Catalysis
4. Product release

Conformational changes in tRNA induced by TruB binding could be detected as hyperchromic shift in tRNA absorption (6), presumably related to detachment of D- and T-arm, and base flipping could be detected by mutating G57 to 2-aminopurine (7). That both processes occur with similar rates (6, 7), implies that they belong to the same step, although step two could potentially involve sub-steps. It is possible but not easily proven, that the U33 label interferes with a part of step 2, which prevents efficient catalysis. A more extensive conformational change affecting remote U33 would at least be better suited to explain the hyperchromic shift, than the detachment of D- and T-arm alone. For pseudouridine formation, it was proposed that the essential aspartate launches either a nucleophilic attack on the C1’ of the respective sugar in a so called ‘acylal mechanism’ (8) (Figure S10 a) or the nucleophile attacks the C5 of the target uridine in a Michael addition (9) (Figure S10 b). The latter mechanism would correspond to the mechanism of several methylgroup transferring enzymes that apply a cysteine residue as nucleophile and that are potently inhibited by 5-fluoro-pyrimidines (10-12).


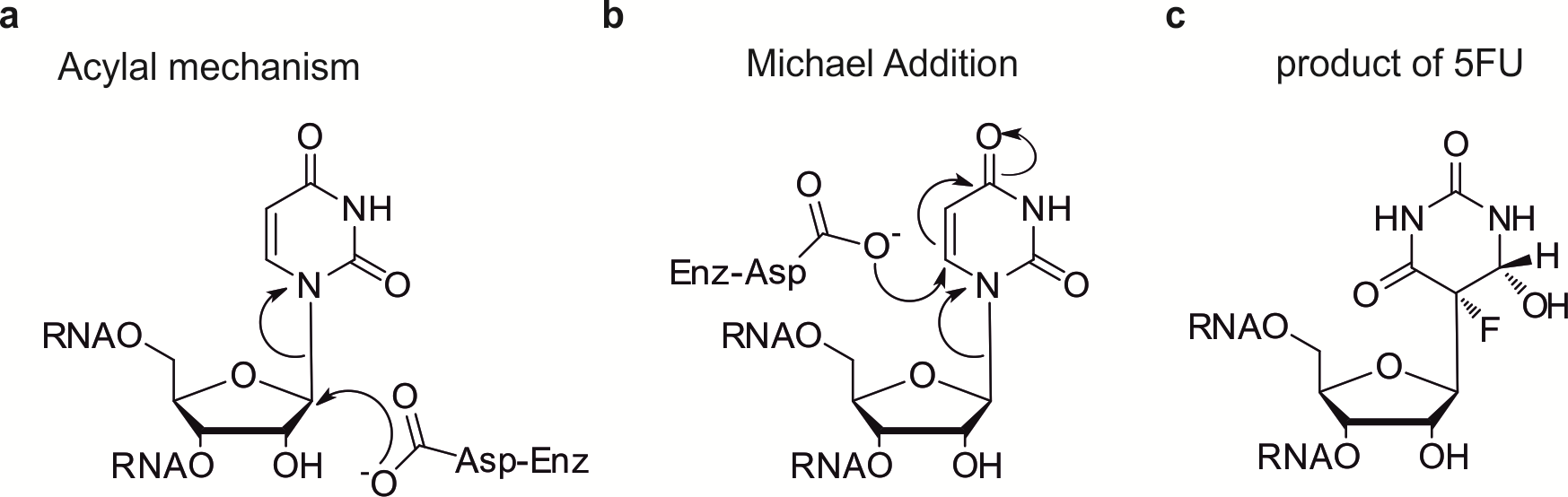


Figure S10. Possible mechanisms of pseudouridine formation: Acylal mechanism (8) (a), Michael addition mechanism (9) (b) and the product of 5FU-turnover by pseudouridine synthases (c).

That 5FU turnover by various pseudouridine synthases yields the rearranged and hydrated compound shown in Figure S10 c, was seen as support to the Michael addition mechanism for a long time, until it could be proven that the hydroxy group does not originate from hydrolysis from hydrolysis of an ester between RNA and enzyme but is rather generated by a nucleophilic attack of water (13). This discovery revealed compound 2 of Figure S11 as last product generated by pseudouridine synthase action on 5FU-RNA.

Remarkably, this product could be generated by either mechanism. A covalent adduct between 5FU-RNA and the catalytic aspartate, which would support a Michael addition-like mechanism, was not reported so far. In contrast, support for an attack on the C1’ accumulates: Recently, Miracco and Mueller characterized the products of TruB action on 5FU-RNA in extensive NMR studies (8). They could show that the enzyme generates two anomeric ribo- (compound 4 of Figure S11) and arabino (compound 5 of Figure S11) products, which is impossible to achieve by a Michael addition-like mechanism (8). Moreover, the inability to detect an arabino-product in turnover of unfluorinated uridine (compound 1 of Figure S11) to pseudouridine (compound 3 of Figure S11) implies that 5FU reacts by another mechanism, than U. Recently a covalent adduct between compound 2 and a tyrosine residue of pseudouridine synthase RIuB, resulting in compound 6, was reported (9). Since compound 2 can result from either mechanism, the existence of compound 6 alone does not favor one mechanism over the other.


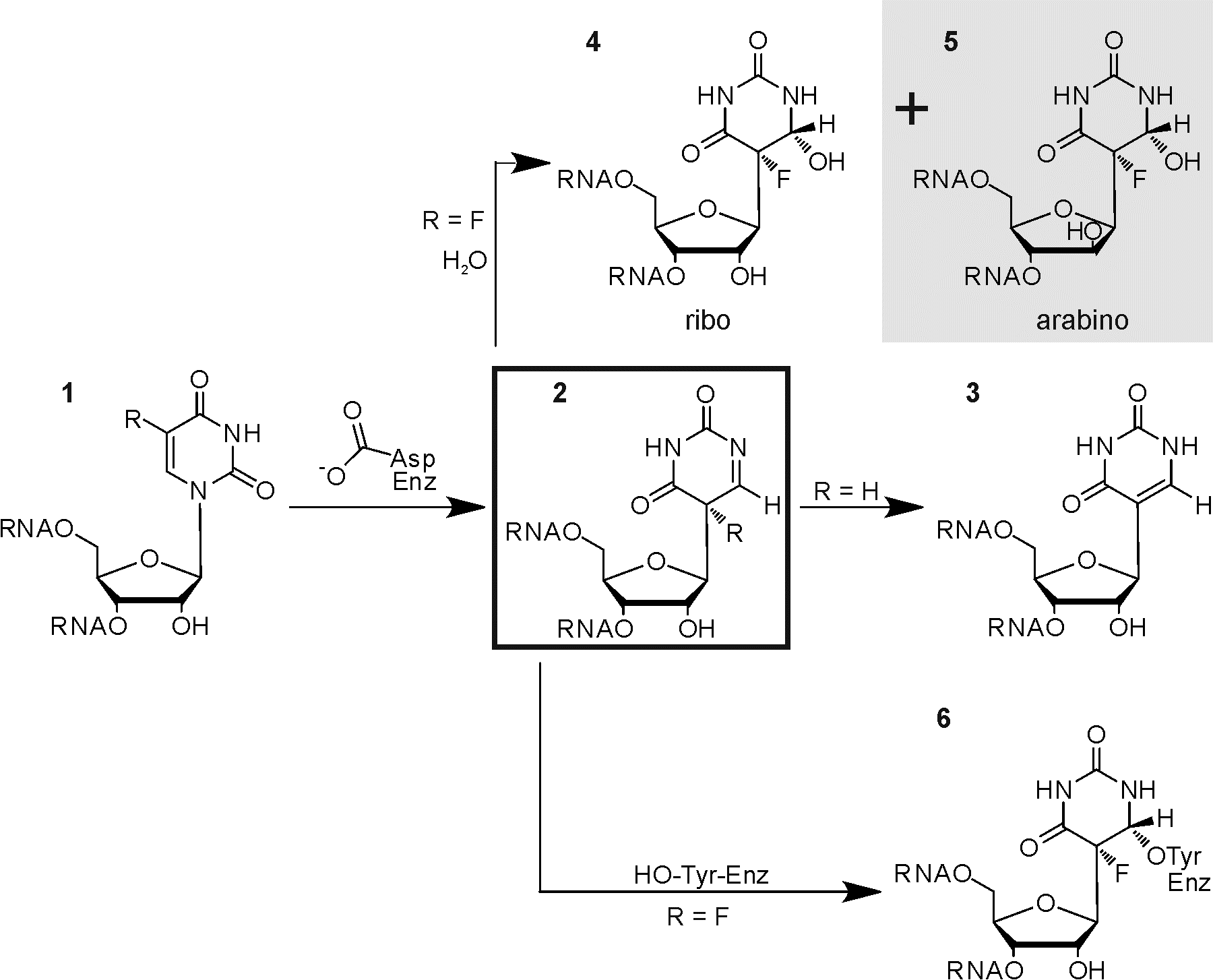


Figure S11. The assumed product of turnover by pseudouridine synthases and the various final products for either 5FU or U turnover (8, 9). The minor arabino product which was only reported until now for 5FU turnover by TruB (8) is shaded in gray, since it cannot be formed from intermediate 2.

Besides by providing an explanation for the existence of compound 5, an attack at C1’ is also plausible, since pseudouridine formation is, formally, a trans-glycosylation reaction: The enzyme family of tRNA-guanine-transglycosylases also applies an aspartate as nucleophile and acts by attacking C1’ (14).

To account for the lack of anomerization in case of pseudouridine formation compared to 5FU turnover, Miracco and Mueller proposed that 5FU reacts by the ‘acylal’ mechanism, while U might react via a third ‘glycal’ mechanism (8). Provided that TruB does not react on 5FU and U by attacking C1’ in the former but C5 in the latter case, a scenario which is not disproven until now and can therefore not be dismissed totally, both mechanisms proposed by Miracco and Mueller are currently the most probable ones. In consequence the ‘gylcal’ mechanism and the ‘acylal’ mechanism as proposed by Miracco and Mueller (8) are depicted in Figure S12.

In the glycal mechanism the glycosidic bond is broken, either first as depicted in Figure S12 a or in concerted fashion with the catalytic aspartate acting as a base at the same time. The aspartate abstracts the proton at C2’, thereby generating the name-giving glycal intermediate, subsequently the free base rotates, gets reattached in a nucleophilic attack and in a final deprotonation step pseudouridine is generated.

For 5FU turnover by TruB Miracco and Mueller proposed an acylal mechanism involving an additional reaction manifold via a glycal intermediate (see Figure S12 b). The catalytic aspartate would attack at the C1’ in concert with or following the resolution of the glycosidic bond, generating the acylal intermediate. Re-substitution of the aspartate by the rotated base might result in direct generation of the major ribo product. Such a mechanism is equally possible for the turnover of U into pseudouridine.

However, Miracco and Mueller offered a reasonable explanation why 5FU turnover could result in two products: In the acylal intermediate of 5FU turnover the base would have a lower nucleophilicity due to the fluorine at C5. This would prolong the lifetime of the acylal intermediate of 5FU, allowing an additional reaction manifold depicted in gray in Figure S12b: Release of the aspartate from the ribose without concomitant attack of the base could allow the aspartate again to deprotonate C2’, generating the glycal intermediate. This intermediate would still have a longer lifetime than its unfluorinated variant, providing the potential of anomerization for 5FU exclusively. Thus, the higher nucleophilicity of the detached base U compared to 5FU can account for the lack of anomerization in pseudouridine formation, independent of whether pseudouridine is generated by an acylal or by a glycal mechanism.


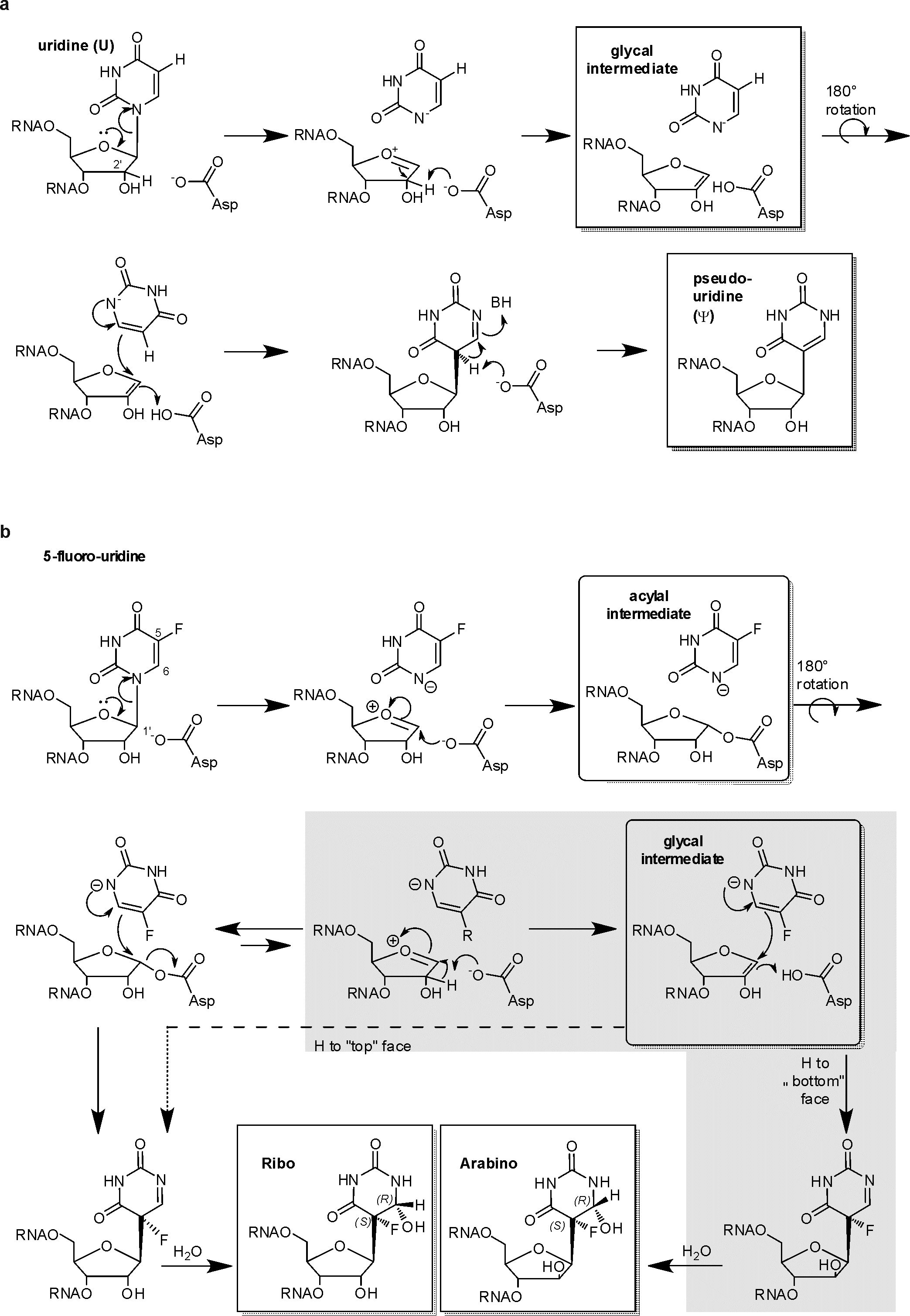


Figure S12. The glycal mechanism for pseudouridine formation as proposed by Miracco and Mueller (8). a In pseudouridine formation the catalytic aspartate acts as a base and generates the glycal intermediate by deprotonating C2’. b In turnover of 5FU the acylal intermediate generated by a nucleophilic attack of the aspartate onto C1’, might possess a prolonged lifetime, since the negative charge in the pyrimidine is stabilized by the fluorine. This would allow an additional, gray shaded reaction manifold to account for the formation of a major ribo- and a minor arabino-product.

Supplementary references:

1. J. R. Lakowicz, (2009) Principles of Fluorescence Spectroscopy., Third edition, Springer
2. Hoang, C.; Ferre-D'Amare, A. R. (2001) Cocrystal structure of a tRNA Psi55 pseudouridine synthase: nucleotide flipping by an RNA-modifying enzyme. *Cell*, **107**, 929-939.
3. Jovine, L, J, Djordjevic, S, Rhodes, D (2000) The Crystal Structure of Yeast Phenylalanine tRNA at 2.0 Å Resolution: Cleavage by Mg^2+^ in 15-year Old Crystals. *J. Mol. Biol.* **301**, 401.414.
4. Pan, H., Agarwalla, S., Moustakas, D. T. Finer-Moore, J. Stroud, R. M. (2003) Structure of tRNA pseudouridine synthase TrUB and its tRNA complex: RNA recognition through a combination of rigid docking and induced fit. *Proc. Natl. Acad. Sci. USA* **100**, 12648-52.
5. Phannachet, K., Huang, R. (2004) Conformational change of pseudouridine 55 synthase upon its association with RNA substrate. *Nucleic Acids Res.*, **32**, 1422-9.
6. Wright, J.R., Keffer-Wilkes, L.C., Dobing, S.R., Kothe, U. (2011) Pre-steady state kinetic analysis of the three *Escherichia coli* pseudouridine synthases TruB, TruA and RIuA reveals uniformly slow catalysis. *RNA*, **17**, 2074-2084.
7. Friedt, J.,Leavens, F.M., Mercier, E., Wieden, H.J., Kothe, U. (2014) An arginine-aspartate network in the active site of bacterial TruB is critical for catalyzing pseudouridine formation. *Nucleic Acids Res.*, **42**, 3857-3870.
8. Miracco, E.J. and Mueller, E.G. (2011) The products of 5-fluorouridine by the action of the pseudouridine synthase TruB disfavor one mechanism and suggest another. *J. Am. Chem. Soc.*, **133**, 11826-11829.
9. Czudnochowski, N., Ashley, G.W., Santi, D.V., Alian, A., Finer-Moore, J. and Stroud, R.M. (2014) The mechanism of pseudouridine synthases from a covalent complex with RNA, and alternate specificity for U2605 versus U2604 between close homologs. *Nucleic Acids Res.*, **42**, 2037-2048
10. Santi, D.V., McHenry, C.S. and Sommer, H. (1974) Mechanism of interaction of thymidylate synthetase with 5-fluorodeoxyuridylate. *Biochemistry*, **13**, 471-481.
11. Santi, D.V. and Hardy, L.W. (1987) Catalytic mechanism and inhibition of tRNA (uracil-5-)methyltransferase: evidence for covalent catalysis. *Biochemistry*, **26**, 8599-8606.
12. Liu, Y., Santi, D.V. (2000) m5C RNA and m5C DNA methyl transferases use different cysteine residues as catalysts *Proc. Natl. Acad. Sci. USA*, **97**, 8263-65.
13. McDonald, M.K., Miracco, E.J., Chen, J., Xie, Y. and Mueller, E.G. (2011) The handling of the mechanistic probe 5-fluorouridine by the pseudouridine synthase TruA and its consistency with the handling of the same probe by the pseudouridine synthases TruB and RluA. *Biochemistry*, **50**, 426-436.
14. Stengl, B., Reuter, K., Klebe, G. (2005) Mechanism and Substrate Specificity of tRNA--Guanine Transglycosylases (TGTs): tRNA-Modifying Enzymes from the Three Different Kingdoms of Life Share a Common Catalytic Mechanism. *Chembiochem*, **6**, 19826-39.
